# Shared metabolomic signatures for prognostic precision across brain injuries

**DOI:** 10.1016/j.bas.2025.105877

**Published:** 2025-11-19

**Authors:** Santtu Hellström, Antti Sajanti, Aditya Jhaveri, Ying Cao, Fredrika Koskimäki, Johannes Falter, Janek Frantzén, Seán B. Lyne, Tomi Rantamäki, Riikka Takala, Jussi P. Posti, Susanna Roine, Sulo Kolehmainen, Bharat Gajera, Kenneth Nazir, Miro Jänkälä, Susanna Piironen, Ahmed Abdirisak, Abhinav Srinath, Romuald Girard, Anni I. Nieminen, Melissa Rahi, Jaakko Rinne, Eero Castrén, Janne Koskimäki

**Affiliations:** a Neurocenter, Department of Neurosurgery, Turku University Hospital and University of Turku, P.O. Box 52, FI-20521, Turku, Finland; b Division of Clinical Neurosciences, Neurosurgery, University of Turku, P.O. Box 52, FI-20521, Turku, Finland; c Neurovascular Surgery Program, Section of Neurosurgery, The University of Chicago Medicine and Biological Sciences, Chicago, IL 60637, USA; d Department of Radiation Oncology, Kansas University Medical Center, Kansas City, KS, 66160, USA; e Neurocenter, Acute Stroke Unit, Turku University Hospital, P.O. Box 52, FI-20521, Turku, Finland; f Department of Neurosurgery, University Medical Center of Regensburg, Regensburg, 93042, Germany; g Department of Neurosurgery, Brigham and Women's Hospital, Harvard Medical School, Boston, MA, USA; h Laboratory of Neurotherapeutics, Drug Research Program, Division of Pharmacology and Pharmacotherapy, Faculty of Pharmacy, University of Helsinki, P.O. Box 56, FI-00014, Helsinki, Finland; i SleepWell Research Program, Faculty of Medicine, University of Helsinki, P.O. Box 63, FI-00014 Helsinki, Finland; j Perioperative Services, Intensive Care and Pain Medicine and Department of Anaesthesiology and Intensive Care, Turku University Hospital and University of Turku, P.O. Box52, FI-20521, Turku, Finland; k Neuroscience Center, HiLIFE, University of Helsinki, P.O. Box 63, FI-00014, Helsinki, Finland; l Helsinki Metabolomics Center, Faculty of Medicine, University of Helsinki, Finland; m Department of Neurosurgery, Oulu University Hospital, Box 25, 90029 OYS, Finland

**Keywords:** Brain injury, Metabolomics, Outcome, Prognosis, Stroke, Traumatic brain injury

## Abstract

**Introduction:**

Metabolomic alterations have been linked to a range of neurological conditions. Investigating temporal changes in serum metabolomic profiles, regardless of the type of brain injury may reveal prognostic indicators.

**Research question:**

We hypothesize that specific metabolomic signatures, conserved across different acute brain injuries, can serve as robust predictors of patient outcomes.

**Material and methods:**

In this longitudinal prospective observational study, serum samples were collected early (2 ± 1 day) and late (6 ± 2 days) post-injury from a total of 73 patients with ischemic stroke (n = 30), aneurysmal subarachnoid hemorrhage (n = 30), and traumatic brain injury (n = 13). Outcomes were categorized as favorable (modified Rankin Scores (mRS) 0–3) and unfavorable (mRS 4–6) three months post-injury. Metabolomic profiling (Orbitrap mass spectrometry) of 462 metabolites, analyzed using statistical and machine learning methods, identified significant outcome differences (p < 0.05, FDR-corrected).

**Results:**

Early-stage samples indicated good prognostic power with a combination of uridine, tryptophan, and lactic acid (AUC 88.8 %, OR 5.29, p < 0.0001). Late-stage samples showed high discriminatory accuracy with a combination of prostaglandin J2, gamma-linolenic acid, N-acetyl-L-alanine, uridine, N-alpha-acetyl-L-asparagine, 3-hydroxy-3-methylglutarate, propionate, and creatinine (AUC 94.4 %, OR 14.5, p < 0.0001). Pathway analyses revealed significant associations with glycolysis/gluconeogenesis, pyrimidine metabolism, and tryptophan metabolism at early stages, and fatty acid biosynthesis, pyruvate metabolism, phenylalanine metabolism, and tryptophan metabolism at later stages.

**Discussion and conclusion:**

These findings underscore the dynamic nature of metabolomic profiles in acute brain injuries and highlight common metabolites as significant prognostic markers across brain injury types.

## Highlights

• Common metabolomic signatures were identified across ischemic stroke, subarachnoid hemorrhage, and traumatic brain injury.
• Conserved metabolites associated with outcome at early and late stages.
• Machine learning–derived prognostic model achieved high accuracy.

## Graphical abstract

**Figure:**
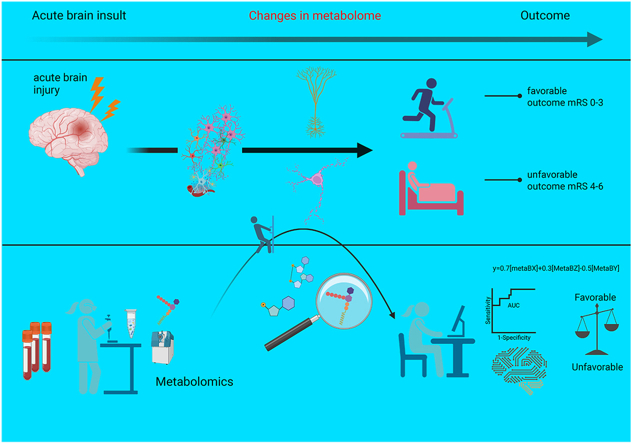

## 1 Introduction

Acute brain injuries (ABIs), including aneurysmal subarachnoid hemorrhage (aSAH), ischemic stroke (IS), and traumatic brain injury (TBI), are major global health concerns, significantly contributing to mortality and disability worldwide (Feigin et al., 2022; Capizzi et al., 2020). Irrespective of etiology, ABIs induce a cascade of substantial secondary cerebral alterations on the cellular, functional and metabolic level that complicate outcome prediction impeding reliable risk stratification and making clinical decision-making and prognostication more difficult (Dagonnier et al., 2021; Maas et al., 2022). A multitude of various metabolites are released into the peripheral blood in response to the initial trauma and downstream pathophysiological reactions, that could be used as metabolomic biomarkers. Altered metabolic pathways are associated with various neurological conditions, including chronic neurodegenerative diseases such as Alzheimer's disease (AD), Parkinson's disease (PD), Huntington's disease (HD), amyotrophic lateral sclerosis (ALS) and multiple sclerosis (MS) (Huo et al., 2020; Shao et al., 2021; Mastrokolias et al., 2016; Goutman et al., 2020; Liu et al., 2022). Metabolic profiles also undergo notable alterations following ABIs (Chi et al., 2021; Shin et al., 2020; Banoei et al., 2023).

Brain injuries involve alterations in excitotoxic or neurotoxic mediators, oxidative stress and corresponding free radicals, lipidomic changes, and shifts in inflammatory mediators (Ricciotti and FitzGerald, 2011; Hajsl et al., 2020; Yan et al., 2015; Sajanti et al., 2024; Hellström et al., 2025). Differentiating factors in ABI metabolomics are detected as varying metabolite concentrations compared to healthy individuals (Sidorov et al., 2019).

In the field of metabolomics, it remains challenging to separate findings that arise directly from the injury from those that reflect secondary responses. Nevertheless, earlier studies have worked to pinpoint pathophysiological shifts in the metabolomic patterns tied to brain injuries (Oft et al., 2024). Excitotoxic metabolites such as glutamate and related amino acids have been observed to play a significant role in the metabolic effects of brain injuries. Studies have found that glutamate and its precursor, glutamine, increase after ABI (Liu et al., 2015; Yang et al., 2017; Tao et al., 2023). This rise is presumably due to increased activity in glial cells aimed at achieving homeostasis following damage (Tao et al., 2023). Additionally, levels of amino acids (AAs) related to glutamine metabolism, proline, and pyroglutamate, have been noted to decrease following a stroke (Sidorov et al., 2019). On the other hand, decreasing glutamine levels have been linked to the severity of stroke (Ahmed et al., 2021). Metabolites that signal oxidative stress, such as lactate, pyruvate, and citrate, change likely due to the initiation of anaerobic energy production. Additionally, levels of valine and isoleucine, which are associated with compensatory energy production methods, have been observed to decrease after ABI (Liu et al., 2015; Dale et al., 2019). After ABIs, the mediators related to cell-mediated inflammation, including phospholipids, and pro-inflammatory cytokines are one important group of metabolites. Phospholipids are the backbone of neuronal cell membranes (Hussain et al., 2020). Phosphatidylethanolamine (PE), phosphatidylcholine (PC), and their hydrolysis products lysophosphatidylethanolamine (LysoPE) and lysophosphatidylcholines (LysoPC) and etherphosphatidylcholines (EPC) have been observed to decrease in patients with ABI (Sidorov et al., 2019; Thomas et al., 2022; Sun et al., 2022). Brain injury activates glutamate-mediated phospholipases A2 and C (PLA2 and PLC), which hydrolyze membrane phospholipids. The resulting decrease in phospholipid levels is accompanied by a concomitant increase in arachidonic acid concentrations following IS (Wieloch and Siesjö, 1982). Reduced concentrations of AAs such as glycine, hippurate, and dimethylamine have been reported in stroke patients. These amino acids are closely linked to folate deficiency and hyperhomocysteinemia, both of which are recognized independent risk factors for stroke. (Jung et al., 2011). In addition, tryptophan is also seen to decrease after ABI because it is connected to the indoleamine-2,3-dioxygenase enzyme which activates after an increase in cytokines (Liu et al., 2015).

Multiple studies have identified changes in the metabolome associated with TBI that correlate with its severity. Using machine learning, Thomas et al. (2022) developed a model consisting of 23 metabolites that effectively distinguished TBI from healthy control patients, independent of the severity of TBI, achieving an area under the curve (AUC) of 0.98 (Thomas et al., 2022). Furthermore, they constructed a model of 19 metabolites to differentiate between three levels of TBI severity. The metabolites encompassed a broad range, including fatty acids (FAs), AAs, and sugar derivatives (Thomas et al., 2022). However, the important discriminative metabolites were three decreased AAs: alanine, threonine, and serine. Additionally, phospholipid levels showed an increasing trend in TBI patients (Thomas et al., 2022). In cases of TBI, significant alterations have been observed in the metabolism of branched-chain amino acids (BCAAs), with most related metabolites predominantly showing a decreasing trend; however, some studies have reported contradictory results (Orešič et al., 2016; Jeter et al., 2013). Additionally, in TBI patients, metabolites within pathways related to glycolysis and FA metabolism primarily exhibit an upward trend compared to healthy patients (Banoei et al., 2023; Thomas et al., 2022; Orešič et al., 2016). Significant metabolic properties have also been identified in the IS group. For instance, increasing purine concentration and decreasing glutamine levels are significantly associated with the severity of stroke (Ahmed et al., 2021; Dale et al., 2019). The prognostic utility of metabolites in ABIs has also been explored. Chi et al. (2021) constructed a model using 63 metabolites, which, when combined with other clinical features, enhanced the predictive capacity for distinguishing outcomes based on the modified Ranking Scale (mRS) (Chi et al., 2021).

This study aims to detect longitudinal metabolomic alterations across brain injuries and identify prognostic biomarkers irrespective of injury type. By highlighting shared metabolic disruptions, the findings may improve outcome prognostication and offer insights into recovery mechanisms, guiding future research and therapeutic strategies.

## 2 Materials and methods

### 2.1 Study design and participants

**Fig. 1:**
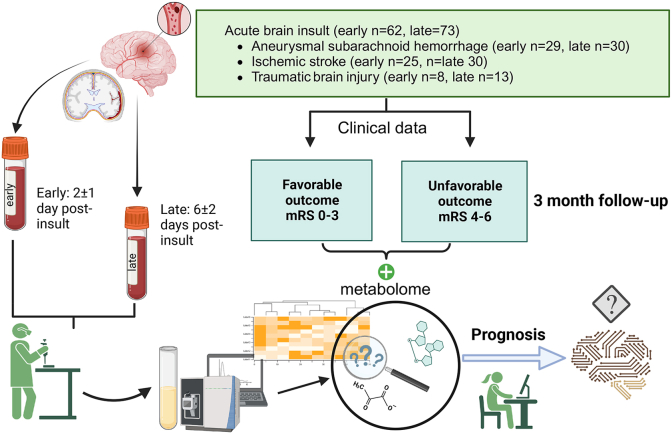
**Flowchart of the study.** In this study, serum samples from patients suffering from aneurysmal subarachnoid hemorrhage, ischemic stroke, and traumatic brain injury were collected at two timepoints post-insult and profiled using the Orbitrap™ platform, capable of identifying 462 metabolites. The importance of these metabolites in predicting patient outcomes was assessed using partial least squares discriminant analysis and t-tests on both early and late samples. The significance of individual metabolites in differentiating outcomes was visualized and further scrutinized with machine learning methodologies. The linear support vector machine algorithm was applied to pinpoint pivotal metabolic features, with performance validated through cross-validation. Potential biomarkers were then evaluated using receiver operating characteristic with LASSO and linear discriminant analysis. Enrichment and pathway analyses were performed to understand pathobiological associations with recovery.

This study was a longitudinal prospective observational cohort design that included 73 consecutive acute brain injury patients from the University Hospital of Turku, Finland, treated between 2016 and 2019 (Fig. 1). These patients were categorized into three groups: ischemic stroke (IS, n = 30), aneurysmal subarachnoid hemorrhage (aSAH, n = 30), and traumatic brain injury (TBI, n = 13) resulting in a subdural hematoma that necessitated surgical intervention.

Eligibility criteria were the diagnosis of A) aSAH, B) IS (embolic, thrombotic, or cryptogenic), or C) TBI leading to an acute subdural hematoma requiring surgical intervention. D) Age above 18 years. E) Provision of informed consent. All patients received standard clinical treatment following the institution's protocols, which align with the prevailing guidelines for treating aSAH, IS, and TBI patients (Connolly et al., 2012; Carney et al., 2017; Powers et al., 2019).

Peripheral venous samples were collected twice from the participants: first, at an early timepoint averaging 2 ± 1 day post-insult, and subsequently, at a later stage averaging 6 ± 2 days post-insult. Three months after the initial event, the aSAH patients had their outcomes assessed during an outpatient clinic visit, while the IS and TBI patient outcomes were assessed through structured telephone interviews. The mRS was employed to evaluate the outcomes, classifying patients as either favorable (mRS 0–3) or unfavorable (mRS 4–6). If a patient died before the 3-month outcome assessment, their mRS was recorded as 6.

During the study recruitment, 11 patients opted out of the study, and one patient initially agreed but later chose to withdraw (late samples cohort: n = 73). Additionally, 11 participants were omitted from the early metabolome biomarker detection measurements because their early samples were not available (early samples cohort n = 62).

### 2.2 Serum extraction

Standard 10 mL BD Vacutainer No Additive collection tubes (REF 364915) were utilized for venous blood serum collection. After drawing the blood, samples were left to rest at room temperature for 30–60 min to facilitate clot formation. Following this clotting period, the samples were centrifuged using a horizontal rotor (swing-out head) at 2200g for 15 min, also at room temperature. The separated serum was then distributed into three clean 10 mL BD Vacutainer No Additive tubes (REF 364915) and stored at −80 °C.

### 2.3 Targeted liquid chromatography-mass spectrometry (LC-MS) metabolomics profiling analytics

A detailed description of the targeted LC-MS metabolomics profiling protocol, including sample preparation, chromatographic separation, and instrument settings, is provided in the Supplementary materials and methods. Briefly, metabolites were quantified using UHPLC-Q-Exactive Orbitrap MS with standardized library references and continuous quality control throughout the analytical run.

## 3 Bioinformatics and statistics

### 3.1 Metabolome data preprocessing

Metabolite data were initially pre-filtered to ensure peak quality, excluding metabolites with poor chromatographic profiles, intensity variation exceeding 20 % RSD in QC samples, or significant carryover. The peak area intensities were normalized by probabilistic quotient normalization to an in-house QC reference using MetaboAnalyst 6.0 (www.metaboanalyst.ca) (Pang et al., 2021). Missing values were imputed at 1/5 of the minimum positive value for each variable. Data underwent log10 transformation and auto-scaling (mean-centered and divided by standard deviation) to standardize metabolite distributions.

### 3.2 Univariate and multivariate analysis

The preprocessed data were analyzed with MetaboAnalyst 6.0 using a range of statistical methods (Xia et al., 2009). For univariate analysis, a *t*-test was applied to identify metabolites significantly associated with patient outcomes (p < 0.05, FDR-corrected). In the multivariate analysis, partial least squares discriminant analysis (PLS-DA) was conducted with 5-fold cross-validation to evaluate model robustness, and cross-validation results included R-squared, Q-squared, and accuracy metrics.

### 3.3 Linear support vector machine to identify features importance

Linear support vector machine (SVM) was employed to identify significant metabolites associated with patient outcomes. Features were selected based on their relative contribution to the classification using cross-validation error rates (10-fold cross-validation) (Xia et al., 2009; Zhang et al., 2006).

### 3.4 Feature selection and combinatory biomarkers

Feature selection for the final models was refined using LASSO regression and K-means clustering (K = 10 clusters) (Tibshirani, 1996). Canonical linear discriminant analysis (LDA) and logistic modeling were applied to develop combinatory biomarkers, predicting patient outcomes with canonical scores (Girard et al., 2018; Srinath et al., 2023). Receiver operating characteristic (ROC) analyses assessed biomarker sensitivity and specificity, with the Youden index determining optimal cutoffs (Youden, 1950).

### 3.5 Quantitative pathway enrichment analysis

To interpret the biological significance of identified metabolites, quantitative pathway enrichment analysis was conducted using the Kyoto Encyclopedia of Genes and Genomes (KEGG) pathways (Kanehisa and Goto, 2000). Pathways differentiating favorable and unfavorable outcomes were identified at both early and late time points. The analysis utilized MetaboAnalyst's quantitative enrichment analysis (QEA) approach, performed with the globaltest package, which applies a generalized linear model to estimate a Q-statistic for each metabolite set (Powers et al., 2019). This Q-statistic reflects the correlation between metabolite concentration profiles and clinical outcomes. For each metabolite set, the Q-statistic is averaged across all metabolites in the set to assess overall pathway significance (Goeman et al., 2004).

### 3.6 Statistical significance

Statistical tests used a significance level of p < 0.05. For analyses involving multiple comparisons, the Benjamini-Hochberg method was applied to control the false discovery rate (FDR) (Benjamini and Hochberg, 1995). Statistical analyses were conducted using MetaboAnalyst 6.0, MetaboAnalyst R (MetaboAnalystR), and SAS (SAS Institute Inc., 2016; Cary, NC, USA).

## 4 Results

### 4.1 Study population and clinical parameters

In the enrolled cohort (n = 73), brain injuries were classified into three primary categories: aSAH represented 41.1 % (30/73), TBI accounted for 17.8 % (13/73) and IS made up 41.1 % (30/73) (Table 1 and Supplemental Table S1). The demographic analysis revealed no significant sex predominance, with males comprising 54.8 % (40/73) of the population (p = 0.12). The mean age of the cohort was 58.3 ± 12.7 years (Table 1). Initial Glasgow Coma Scale (GCS) assessments at the scene recorded a mean score of 11.8 ± 4.4, with scores ranging from 3 to 15 (Supplemental Table S1). Out of the enrolled cohort (n = 73), 11 early samples were unavailable, leaving 62 samples for the early cohort. The early (n = 62) and late (n = 73) sample cohorts were assessed for balanced grouping concerning age, sex, type of brain injury, and mRS level (p = 0.98, p = 0.12, p = 0.68, and p = 0.69, respectively) (Table 1).

**Table 1:** **Basic characteristics of early and late cohorts.** Favorable modified Rankin scale (mRS) 0–3, Unfavorable mRS 4–6. aSAH = aneurysmal subarachnoid hemorrhage, TBI = traumatic brain injury, IS = ischemic stroke.

Variables	Early (n = 62)	Late (n = 73)	p-value
**Age in years**			0.98
Mean ± SD	58.3 ± 13.1	58.3 ± 12.7	
Min–Max	23.0–75.0	23.0–75.0	
Median (IQR)	63.5 (47.0–70.0)	61.0 (47.5–75.0)	
**Sex**			0.12
Male (%)	33 (53.2)	40 (54.8)	
Female (%)	29 (46.8)	33 (45.2)	
**Type of brain injury**			0.68
aSAH (%)	29 (46.8)	30 (41.1)	
TBI (%)	8 (12.9)	13 (17.8)	
IS (%)	25 (40.3)	30 (41.1)	
**mRS**			0.69
Favorable (%)	37 (59.7)	40 (54.8)	
Unfavorable (%)	25 (40.3)	33 (45.2)	

Statistical comparisons to detect group differences between early and late study cohorts (all comparisons p > 0.05). Unpaired two-sample *t*-test (continuous) or Chi-square test (categorical) for p-values.

Outcomes were assessed at the three-month mark using the mRS. Favorable outcomes (mRS scores 0–3) were recorded in 54.8 % (40/73) of the patients, while unfavorable outcomes (mRS scores 4–6) were observed in 45.2 % (33/73) (Table 1). The overall mortality rate within the cohort at three months post-injury was 19.2 % (14/73). Additionally, the favorable (n = 45) and unfavorable (n = 28) sub-cohort groups were balanced concerning age, sex, and type of brain injury (p = 0.21, p = 0.52, and p = 0.09, respectively) (Table 2).

**Table 2:** **Basic characteristics of favorable and unfavorable patient groups in late time point (n = 73).** Modified Rankin scale (mRS). Favorable mRS 0–3, Unfavorable mRS 4–6. aSAH = aneurysmal subarachnoid hemorrhage, TBI = traumatic brain injury, IS = ischemic stroke.

Variables	Favorable (n = 45)	Unfavorable (n = 28)	p-value
**Age in years**			0.21
Mean ± SD	56.8 ± 12.4	60.6 ± 13.2	
Min–Max	23.0–75.0	30.0–74.0	
Median (IQR)	59.0 (47.0–66.5)	65.0 (50.0–71.0)	
**Sex**			0.52
Male (%)	26 (57.8)	14 (50.0)	
Female (%)	19 (42.2)	14 (50.0)	
**Type of brain injury**			0.09
aSAH (%)	18 (40.0)	12 (42.8)	
TBI (%)	5 (11.1)	8 (28.6)	
IS (%)	22 (48.9)	8 (28.6)	

Statistical comparisons to detect group differences between favorable and unfavorable outcome groups. Two-sample *t*-test (continuous) or Chi square test exact test (categorical) for p-values.

### 4.2 Metabolomic profiles of acute brain injuries

**Fig. 2:**
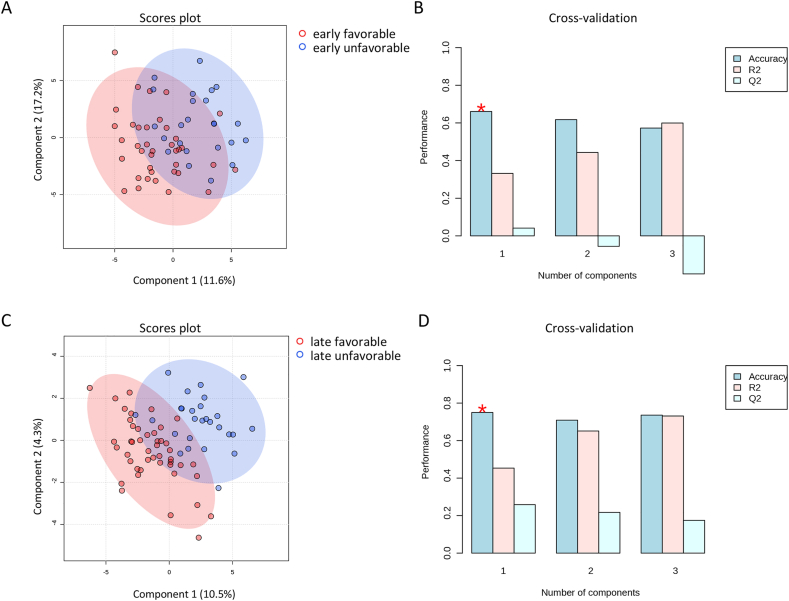
**Partial least squares discriminant analysis (PLS-DA) and cross-validation of the identified serum metabolome. A and B**) The early serum samples (2 ± 1 day post-insult) indicated limited overall discriminatory ability; the low Q2 value in cross-validation suggests slight overfitting of the model (accuracy = 0.66, R2 = 0.33, Q2 = 0.04). **C and D**) The late serum samples (6 ± 2 days post-insult) cohort showed good discriminatory ability confirmed by cross-validation (accuracy = 0.79, R2 = 60.0, Q2 = 0.24).

The concentrations of circulating metabolites were quantitatively measured in serum samples at two timepoints (early = 2 ± 1 day and late = 6 ± 2 days). The normalized serum metabolome, which included 462 metabolites screened, revealed significant metabolic changes. We specifically analyzed both early (n = 62) and late (n = 73) sample cohorts. Analysis of early serum samples showed limited discriminatory ability between favorable and unfavorable outcomes during cross-validation (accuracy = 0.66, R-squared = 0.33, Q-squared = 0.04) (Fig. 2A and B). Conversely, the late serum samples displayed improved discriminatory potential with good cross-validation performance (accuracy = 0.79, R-squared = 60.0, Q-squared = 0.24) (Fig. 2C and D). We also analyzed each disease group within the same outcome categories and timepoints. None of the diseases clustered separately (Supplemental Fig. 1).

Favorable outcome = modified Rankin Scale (mRS) 0–3; unfavorable outcome = mRS 4–6. ∗ indicates highest accuracy. Early timepoint: n = 62; late timepoint: n = 73. R^2^ = R-squared; Q^2^ = cross-validated R-squared.

### 4.3 Metabolomic profiles associated with patient outcomes across acute brain injuries

**Fig. 3:**
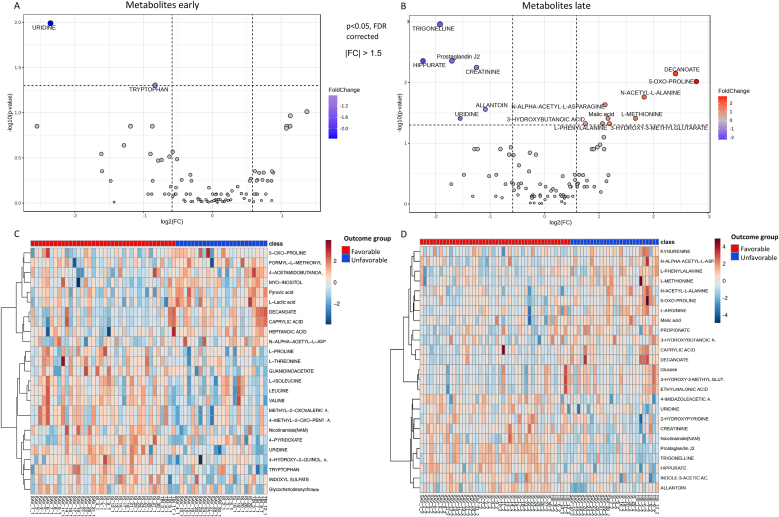
**Metabolome signatures differentiating patients with favorable and unfavorable outcomes after acute brain. A**) A volcano plot analyzing early samples showing statistically significant metabolites (up 0, down 2) differentiating favorable and unfavorable outcome groups (p < 0.05, FDR-corrected, |FC| > 1.5). **B**) A volcano plot analyzing late samples showing statistically significant metabolites (up 9, down 6) differentiating favorable and unfavorable outcome groups (p < 0.05, FDR-corrected, |FC| > 1.5). **C**) Hierarchical clustering heatmap identifying the top 25 metabolites in the early cohort. Distance measure: Euclidean distance. Statistical measure: *t*-test. Separation patterns per fold change (red increased, blue decreased) of the favorable and unfavorable groups are observed in the heatmap. **D**) Hierarchical clustering heatmap identifying the top 25 metabolites in the late cohort. Distance measure: Euclidean distance. Statistical measure: *t*-test. Separation patterns per fold change (red increased, blue decreased) of the favorable and unfavorable groups are observed in the heatmap.

Temporal alterations in the metabolome following different brain injuries were evident (Fig. 3A–D). We analyzed the presence of metabolites that significantly differentiated between the two outcome groups. Early samples revealed only two statistically significant metabolites—uridine and tryptophan—that differentiated favorable from unfavorable outcomes (p < 0.05, FDR-corrected) (Fig. 3A–Supplemental Table S2). Lactic acid showed a trend towards significance (p < 0.1, FDR-corrected). However, in the late sample group, 15 metabolites were identified as statistically significant for differentiating outcomes, including prostaglandin J2 (PGJ2), creatinine, N-acetyl-L-alanine, L-methionine, uridine, N-alpha-acetyl-L-asparagine, allantoin, malic acid, 3-hydroxy-3-methylglutarate, 3-hydroxybutanoic acid, L-phenylalanine, 5-oxo-proline, trigonelline, decanoate, and hippurate (p < 0.05, FDR-corrected) (Fig. 3B–Supplemental Table S3). Hierarchical clustering heatmaps identified the top 25 metabolites for each cohort, illustrating distinct separation patterns between the favorable and unfavorable groups (red indicating increased and blue indicating decreased metabolites) (Fig. 3C and D).

### 4.4 Assessing single metabolomic predictors of patient outcomes

**Fig. 4:**
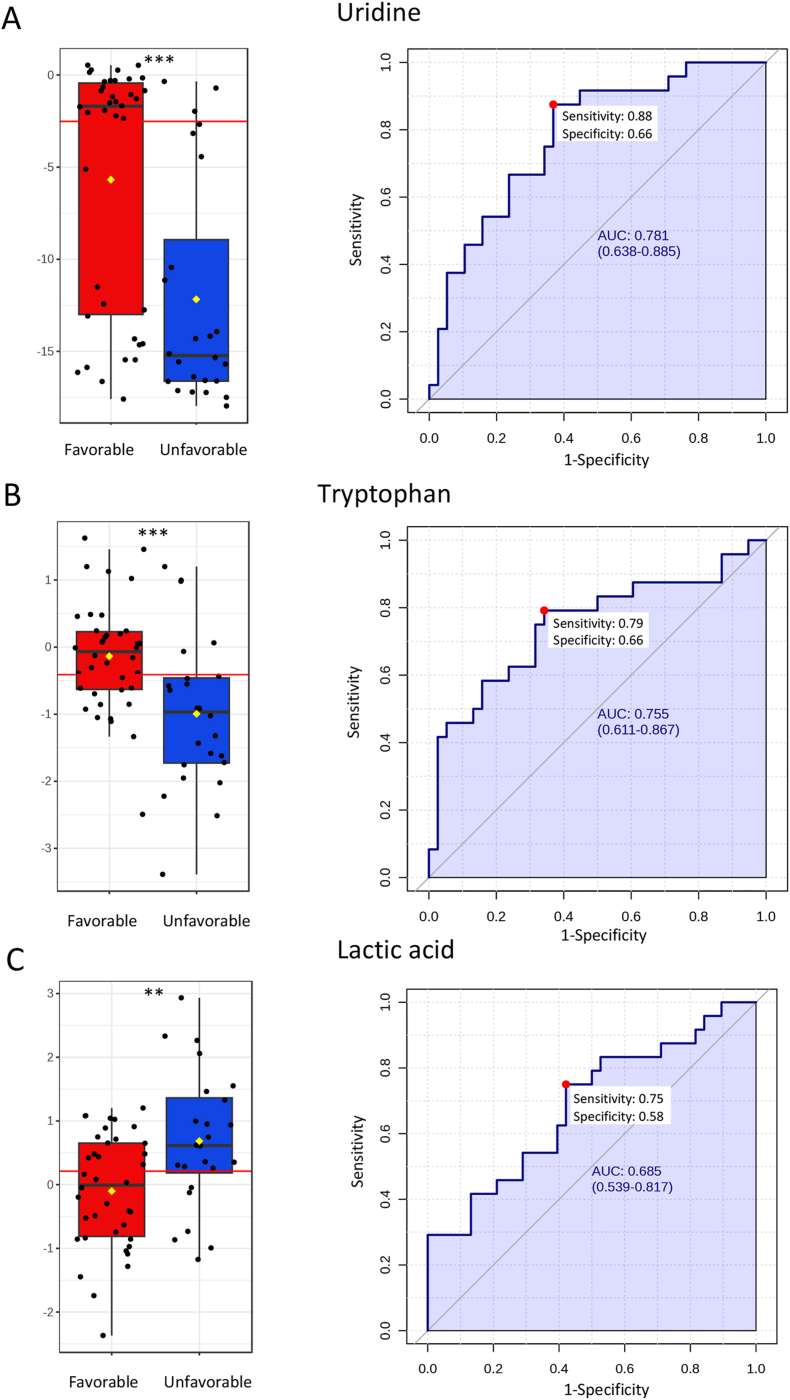
**Univariate and the receiver operating characteristic (ROC) analyses for early metabolites. A**) The best performing early metabolite uridine (p < 0.0001) differentiated favorable outcome patients from unfavorable outcome patients with 88 % sensitivity and 66 % specificity (area under the curve (AUC) 78.1 % with 95 % confidence interval (CI) = 64–89 %). **B**) The second best performing early metabolite tryptophan (p < 0.0001) differentiated favorable outcome patients from unfavorable outcome patients with 79 % sensitivity and 66 % specificity (AUC 75.5 % with 95 % CI = 61–87 %). **C**) Lactic acid (p = 0.0029) identified using the LASSO method differentiated favorable outcome patients from unfavorable outcome patients with 75 % sensitivity and 58 % specificity (AUC 68.5 % with 95 % CI = 54–82 %). Box plot presented ± IQR, yellow diamond indicates mean concentration, horizontal red line indicates the optimal cut-off. Optimal cut-off point for the prognostic test was determined by calculating the Youden index (red dot in AUC curve). The 95 % confidence intervals were calculated using 500 bootstrappings. Box plot y-axis = normalized concentration with batch correction, x-axis = favorable (red) and unfavorable outcome (blue) groups. ∗∗∗p < 0.0001. ∗∗p = 0.003.

ROC analyses were applied to assess the metabolome for potential biomarkers (Fig. 4). In the early samples, the best-performing metabolite was uridine, which distinguished favorable outcome patients from unfavorable outcome patients with 88 % sensitivity and 66 % specificity (AUC 78.1 %, 95 % CI: 64–89 %, p < 0.0001) (Fig. 4A). The second best performing early metabolite, tryptophan, demonstrated 79 % sensitivity and 66 % specificity (AUC 75.5 %, 95 % CI: 61–87 %, p < 0.0001) (Fig. 4B). Lactic acid was identified as an important candidate using the LASSO method (100 % frequency) and with a trend towards significance in initial analysis (p < 0.05, FDR-corrected), showed 75 % sensitivity and 58 % specificity (AUC 68.5 %, 95 % CI: 54–82 %, p = 0.0029) (Fig. 4C).

**Fig. 5:**
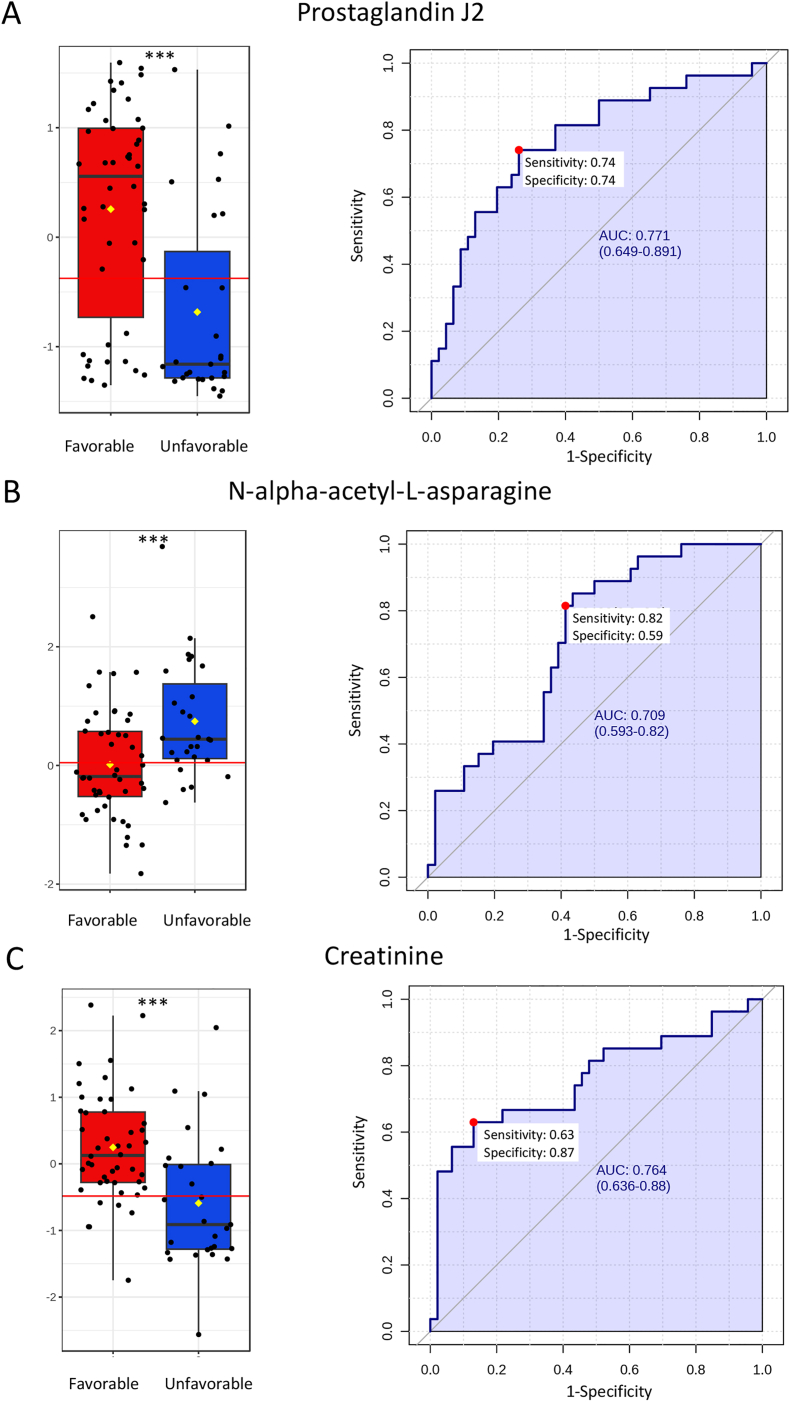
**Univariate and the receiver operating characteristic (ROC) analyses for three best-performing metabolites identified with LASSO in late point. A**) The best performing late metabolite prostaglandin J2 (p < 0.0001) differentiated favorable outcome patients from unfavorable outcome patients with 74 % sensitivity and 74 % specificity (area under the curve (AUC) 77.1 % with 95 % confidential interval (CI) = 65–89 %). **B**) Metabolite N-alpha-acetyl-L-asparagine (p < 0.0001) differentiated favorable outcome patients from unfavorable outcome patients with 82 % sensitivity and 59 % specificity (AUC 70.1 % with 95 % CI = 59–82 %). **C**) Creatinine (p < 0.0001) differentiated favorable outcome patients from unfavorable outcome patients with 63 % sensitivity and 87 % specificity (AUC 76.4 % with 95 % CI = 64–88 %). Box plot presented ± IQR, yellow diamond indicates mean concentration, horizontal red line indicates the optimal cut-off. Optimal cut-off point for the prognostic test was determined by calculating the Youden index (red dot in the AUC curve). The 95 % confidence intervals were calculated using 500 bootstrappings. Box plot y-axis = normalized concentration with batch correction, x-axis = favorable (red) and unfavorable outcome (blue) groups. ∗∗∗p < 0.0001.

For the late samples, PGJ2 emerged as the top metabolite, differentiating favorable from unfavorable outcomes with 74 % sensitivity and 74 % specificity (AUC 77.1 %, 95 % CI: 65–89 %, p < 0.0001) (Fig. 5A). N-alpha-acetyl-L-asparagine was the second best, with 82 % sensitivity and 59 % specificity (AUC 70.1 %, 95 % CI: 59–82 %, p < 0.0001) (Fig. 5B). Creatine showed 63 % sensitivity and 87 % specificity (AUC 76.4 %, 95 % CI: 64–88 %, p < 0.0001, FDR-corrected) (Fig. 5C). These metabolites demonstrated substantial potential as prognostic biomarkers, providing significant insights into patient outcomes across different acute brain injuries.

### 4.5 Multivariate machine learning for outcome discrimination

To handle the high-dimensional data typical in metabolomics studies, machine learning techniques (untargeted linear SVM approach) were employed to identify significant metabolic features that differentiate patients with favorable outcomes from those with unfavorable outcomes at both early and late time points.

**Fig. 6:**
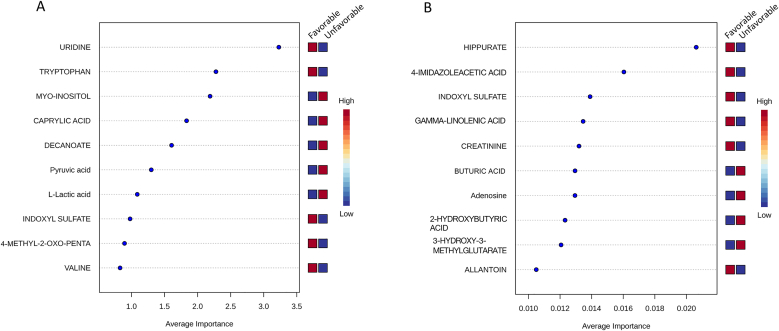
**Untargeted linear support vector machine (SVM) algorithm analysis to identify important metabolic features. A**) Identified important metabolic features showing the top 10 metabolites (according to importance in SVM models) differentiating the favorable outcome patients from unfavorable outcome patients at the early time point. **B**) Identified important metabolic features showing the top 10 metabolites (according to importance in SVM models) differentiating the favorable outcome patients from unfavorable outcome patients at the late timepoint.

The analysis highlighted the top 10 metabolites for each time point based on their importance in the models. At the early time point, key metabolites included valine, 4-methyl-2-oxo-pentanoate, indoxyl sulfate, L-lactic acid, pyruvic acid, decanoate, caprylic acid, myo-inositol, tryptophan, and uridine (Fig. 6A).

For the late time point, significant metabolites identified were allantoin, 3-hydroxy-3-methylglutarate, 3-hydroxybutanoic acid, adenosine, butyric/isobutyric acid, creatinine, gamma-linolenic acid (GLA), indoxyl sulfate, 4-imidazoleacetic acid, and hippurate (Fig. 6B). These late-stage metabolites were important in differentiating the outcome groups, suggesting that the metabolic profile evolves over time.

### 4.6 Combinatory biomarker analysis through machine learning LDA

**Fig. 7:**
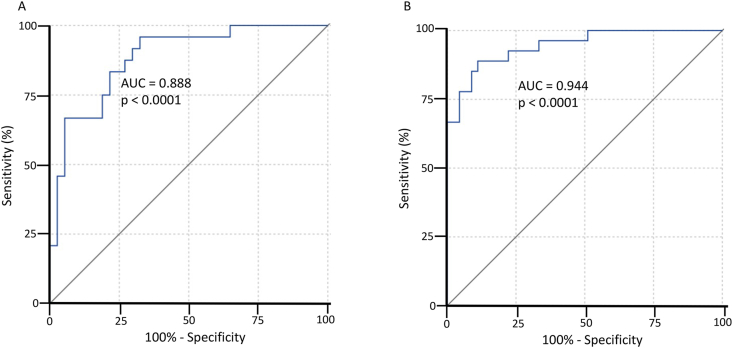
**Linear discriminant analyses (LDA) of the selected candidate biomarkers. A**) In the early set, the receiver operating characteristic curve (ROC) of three identified candidate metabolites (uridine, tryptophan, and lactic acid) in LDA prognosing favorable outcome: odds ratio (OR) 5.29 (95 % confidence interval (CI) 2.30–12.0); Area under the curve (AUC) = 88.8 %, 95 % CI = (80–97 %), p < 0.0001, with 71 % sensitivity and 92 % specificity J = 0.63). LDA of these three metabolites resulted an equation with canonical scores: 0.766[Uridine] + 0.490[Tryptophan] – 0.561[Lactic acid] **B**) In the late set, the ROC of eight identified candidate metabolites (prostaglandin J2, gamma-linolenic acid, N-acetyl-L-alanine, uridine, N-alpha-acetyl-L-asparagine, 3-hydroxy-3-methylglutarate, propionate and creatinine) in LDA prognosing favorable outcome: OR 14.5 (95 % CI 3.93–52.6); area under the curve (AUC) = 94.4 %, 95 % CI = (89–100 %), p < 0.0001, with 89 % sensitivity and 89 % specificity (J = 0.78). LDA of these 8 metabolites resulted an equation with canonical scores: 0.203[Prostaglandin J2] – 0.568[Gamma-linolenic acid] – 0.178[N-acetyl-L-alanine] + 0.148[Uridine] – 0.581[N-alpha-acetyl-L-asparagine] – 0.419[3-hydroxy-3-methylglutarate] – 0.460[Propionate] + 0.903[Creatinine]. Optimal cut-off point for the prognostic test was determined by calculating the Youden index (J).

LDA was employed to develop combinatory biomarker models, effectively integrating multiple metabolites to enhance prognostic accuracy. In the early sample cohort, we identified combinatory biomarkers candidates with the LASSO regression method with 100 % Lasso frequency for lactic acid, tryptophan, and uridine. Subsequent K-means (KM) clustering (K = 10) identified that each of the 3 metabolites clustered differently. The LDA model incorporating these three candidate metabolites demonstrated robust prognostic capability, with an AUC of 88.8 % (95 % CI: 80–97 %, p < 0.0001), 71 % sensitivity, and 92 % specificity (J = 0.63) (Fig. 7A). The corresponding LDA equation was formulated as:

Canonical score = 0.766[Uridine] + 0.490[Tryptophan] – 0.561[Lactic acid].

This equation differentiated favorable outcomes with an odds ratio (OR) of 5.29 (95 % CI: 2.30–12.0).

In the late metabolome, we again employed LASSO regression and KM clustering (K = 10 clusters). Seven late metabolites demonstrated distinct clustering behavior in the analysis with selection frequencies 100-30 % (gamma-linolenic acid = 30 %, N-acetyl-L-alanine = 90 %, uridine = 80 %, N-alpha-acetyl-L-asparagine = 60 %, 3-hydroxy-3-methylglutarate = 30 %, propionate = 30 % and creatinine = 100 %). PGJ2 had 0 selection frequency but this metabolite was added to the final model due to high AUC (Fig. 5). This combinatory model achieved an AUC of 94.4 % (95 % CI: 89–100 %, p < 0.0001), with both sensitivity and specificity at 89 % (J = 0.78) (Fig. 7B). The LDA equation for this late-stage model was formulated as:

*Canonical score = 0.203[Prostaglandin J2] – 0.568[Gamma-linolenic acid] – 0.178[N-acetyl-L-alanine] + 0.148[Uridine] – 0.581[N-alpha-acetyl-L-asparagine] – 0.419[3-hydroxy-3-methylglutarate] – 0.460[Propionate] + 0.903[Creatinine]*.

This equation differentiated outcomes with an OR of 14.5 (95 % CI: 3.93–52.6).

To further enhance predictive accuracy, we investigated the impact of combining early and late prognostic information through LDA. The combinatory model yielded a high AUC of 95.8 % (95 % CI: 91–100 %, p < 0.0001), with 87 % sensitivity and 97 % specificity, and an odds ratio (OR) of 12.5 (95 % CI: 3.27–47.6) (Supplemental Fig. S2).

## 5 Functions of the identified metabolites

### 5.1 Early time point quantitative pathway enrichment analysis

**Fig. 8:**
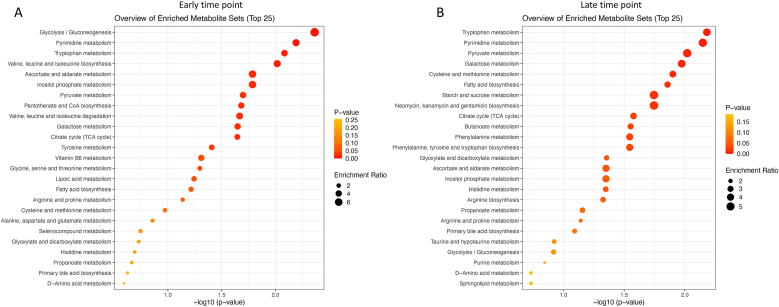
**KEGG quantitative pathway enrichment analysis of metabolic changes at early (A) and late (B) time points differentiating favorable and unfavorable outcomes following acute brain injury. A)** At the early time point, 11 pathways approached significance at p < 0.1, FDR-corrected. Pathways associated with immediate energy production and essential metabolic processes were prominent, including Glycolysis/Gluconeogenesis (p = 0.0043, FDR = 0.088), Inositol Phosphate metabolism (p = 0.0163, FDR = 0.088), and Pantothenate and CoA biosynthesis (p = 0.0208, FDR = 0.088). **B)** At the late time point, seven pathways were close to significance at p < 0.1, FDR-corrected. Pathway analysis showed a shift toward long-term metabolic adaptations, with pathways including Fatty Acid biosynthesis (p = 0.0139, FDR = 0.097), Cysteine and Methionine metabolism (p = 0.0126, FDR = 0.097), and Starch and Sucrose metabolism (p = 0.0180, FDR = 0.097). Tryptophan metabolism (early p = 0.0082, FDR = 0.088; late p = 0.0066, FDR = 0.097) was significant at both time points.

At the early time point, quantitative pathway enrichment analysis identified several metabolic pathways with potential differences between favorable and unfavorable outcomes in acute brain injuries (Fig. 8A, Supplemental Table S4). Even though raw p-values showed robust effect sizes for a number of metabolic pathways, due to the small sample size and at the same time high number of comparisons FDR-corrected significance threshold of p < 0.05 was missed ubiquitously. Yet, potentially pathways including Glycolysis/Gluconeogenesis (p = 0.0043, FDR = 0.088), Pyrimidine metabolism (p = 0.0064, FDR = 0.088), Tryptophan metabolism (p = 0.0082, FDR = 0.088), and Valine, Leucine, and Isoleucine biosynthesis (p = 0.0096, FDR = 0.088) showed a promising differential signal between groups. Additional pathways with significant uncorrected p-values were Ascorbate and Aldarate metabolism, Inositol Phosphate metabolism, Pyruvate metabolism, Pantothenate and CoA biosynthesis, Valine, Leucine, and Isoleucine degradation, Galactose metabolism, and the Citrate cycle (TCA cycle), yet, FDR-corrected p-value was 0.088.

### 5.2 Late time point quantitative pathway enrichment analysis

At the late time point, quantitative pathway enrichment analysis identified several metabolic pathways that may differ between favorable and unfavorable outcomes in acute brain injury (Fig. 8B–Supplemental Table S4). Although no pathways reached statistical significance after FDR correction (FDR <0.05), several exhibited uncorrected p-values suggestive of potential biological relevance. These included Tryptophan metabolism (p = 0.0066, FDR = 0.097), Pyrimidine metabolism (p = 0.0071, FDR = 0.097), Pyruvate metabolism (p = 0.0095, FDR = 0.097), Galactose metabolism (p = 0.0106, FDR = 0.097), Cysteine and Methionine metabolism (p = 0.0126, FDR = 0.097), Fatty Acid biosynthesis (p = 0.0139, FDR = 0.097), and Starch and Sucrose metabolism (p = 0.0180, FDR = 0.097). Additional pathways with FDR values around 0.10 included the Citrate cycle (TCA cycle), Butanoate metabolism, Phenylalanine metabolism, and Phenylalanine, Tyrosine, and Tryptophan biosynthesis. While these findings do not meet conventional thresholds for statistical significance, they suggest potential shifts in metabolic processes over time following acute brain injury, warranting further investigation.

### 5.3 Differences between early and late pathways

The pathway analyses demonstrate dynamic shifts in metabolic processes as the response to acute brain injury progresses (Fig. 8). At early time points, significant enrichment was observed in pathways such as glycolysis/gluconeogenesis (p = 0.0043, FDR = 0.088), inositol phosphate metabolism (p = 0.0163, FDR = 0.088), and pantothenate and CoA biosynthesis (p = 0.0208, FDR = 0.088), highlighting immediate metabolic adaptations to injury. In contrast, the late phase was characterized by enrichment of pathways involved in fatty acid biosynthesis (p = 0.0139, FDR = 0.097), cysteine and methionine metabolism (p = 0.0126, FDR = 0.097), and starch and sucrose metabolism (p = 0.0180, FDR = 0.097), indicating a shift toward longer-term metabolic remodeling. Notably, tryptophan metabolism was consistently enriched at both early (p = 0.0082, FDR = 0.088) and late (p = 0.0066, FDR = 0.097) time points, suggesting its sustained involvement throughout the injury response.

## 6 Discussion

The findings from our prospective cohort study indicate that identifying shared circulating blood metabolites can significantly enhance prognostication of outcomes in ABI patients. Prognostic accuracy is evident at both early and late time points, with greater predictive strength observed in later stages when more significant metabolites are incorporated into the model. Our study adds to the existing literature by leveraging machine learning to develop metabolite-based models for predicting ABI outcomes, with distinct models based on early-phase, late-phase, and combined metabolite data. This research identifies key biomarkers and associated metabolic pathways, and emphasizes notable temporal metabolic shifts following brain injury, potentially guiding future clinical interventions and therapeutic strategies.

### 6.1 Comparison of metabolite model with previously developed models

Our study's metabolite models for early (AUC 88.8 %) and late (AUC 94.4 %) time points demonstrate robust prognostic performance, comparable to or exceeding prior models developed for specific ABI types. For instance, Thomas et al. (2022) reported an AUC of 0.83 for a 19-metabolite model predicting TBI outcomes, emphasizing AAs (e.g., alanine, threonine, serine) and phospholipids (Thomas et al., 2022). Similarly, Oresic et al. (2016) achieved an AUC of 0.84 with a 49-metabolite model for TBI, highlighting medium-chain fatty acids (e.g., octanoic acid, decanoic acid) and sugar derivatives (Orešič et al., 2016). For IS, Chi et al. (2021) developed a 63-metabolite model with enhanced predictive capacity when combined with clinical features, though specific AUC values were not isolated for metabolites alone (Chi et al., 2021).

Our early model, relying on just three metabolites—uridine, tryptophan, and lactic acid—achieves a high AUC while using only a few key metabolites. In contrast to the larger metabolite panels reported in previous studies, this efficient model suggests that a focused set of biologically relevant markers may be sufficient to capture critical prognostic information during the acute phase across different types of acute brain injury. TÄHÄN Uridine's prominence aligns with its neuroprotective roles noted in preclinical TBI and neurodegenerative models (Dempsey and Raghavendra Rao, 2003; Kabadi and Maher, 2010), though its prognostic utility in humans has been underexplored. Tryptophan's inclusion aligns with prior stroke studies linking kynurenine pathway dysregulation to outcome severity (Hajsl et al., 2020; Mo et al., 2014), while lactic acid's role as an energy metabolism marker is consistent with findings in TBI and aSAH (Thomas et al., 2022; Dijkland et al., 2015).

The late model, incorporating eight metabolites, outperforms many prior models in predictive accuracy (AUC 94.4 %) and reflects a broader metabolic scope. Unlike Thomas et al.’s (2022) focus on amino acids and phospholipids or Oresic et al.’s (2016) emphasis on fatty acids, our late model integrates diverse classes—lipids (PGJ2, GLA), amino acid derivatives (N-acetyl-L-alanine, N-alpha-acetyl-L-asparagine), and energy metabolites (creatinine, propionate)—potentially capturing a more holistic metabolic response. The inclusion of PGJ2, with its dual pro- and anti-inflammatory roles (Liu et al., 2013; Zhao et al., 2006), and GLA, with its anti-inflammatory properties (Sergeant et al., 2016), introduces novel prognostic candidates not prominently featured in earlier ABI models.

Importantly, our models, based on substantially fewer metabolites than previously reported, still show strong prognostic performance across aSAH, IS, and TBI, suggesting their potential utility despite the heterogeneous pathophysiology of ABI. The reduced number of metabolites brings such models closer to clinical applicability. In the future, incorporation of metabolomic profiling into clinical practice—alongside radiological and neurological assessments—could enable more accurate early prognosis and support more personalized rehabilitation strategies. Validation in larger, multicenter cohorts will be essential to confirm these findings.

A key distinction of our study is its pan-ABI approach, encompassing IS, aSAH, and TBI, rather than focusing on a single injury type. This heterogeneity likely broadens the detected metabolic signatures, as evidenced by the lack of disease-specific clustering, suggesting shared pathophysiological metabolic response, and reflected mechanisms across ABI types. While prior models often achieved high specificity within a single condition, our cross-injury approach sacrifices some specificity for generalizability, offering a unified metabolomic framework applicable to diverse clinical settings.

### 6.2 Temporal changes in the enriched metabolomic pathways

Our study delineates distinct temporal shifts in metabolomic pathways, mirroring the dynamic pathophysiology of ABI across early and late time points. At the early stage, enriched pathways—glycolysis/gluconeogenesis, pyrimidine metabolism, and tryptophan metabolism—reflect an acute metabolic crisis marked by energy deficits and neuroinflammation. The early enrichment of glycolysis and gluconeogenesis likely reflects impaired oxidative metabolism and a compensatory shift toward anaerobic ATP production after ABI. Elevated lactic acid, a key feature in our early model distinguishing favorable from unfavorable outcomes, supports this mechanism, as lactate is the primary end-product of anaerobic glycolysis and indicates disrupted cerebral energy homeostasis (Dijkland et al., 2015; Svedung Wettervik et al., 2020). These mechanistic patterns highlight early energy failure as a potential target for future therapeutic studies. Tryptophan metabolism's prominence highlights early neuroinflammatory cascades via the kynurenine pathway, where increased kynurenine/tryptophan ratios correlate with worse stroke outcomes (Brouns et al., 2010; Gold et al., 2011), aligning with our findings of sustained inflammatory stress.

At the late stage, pathways such as fatty acid biosynthesis, pyruvate metabolism, and phenylalanine metabolism emerge, alongside persistent tryptophan metabolism enrichment. This shift suggests a transition from acute energy stress to adaptive biosynthetic and inflammatory resolution processes. Metabolites like GLA and PGJ2, lower and higher respectively in favorable outcomes, indicate a balance between pro- and anti-inflammatory lipid mediators, supporting lipid metabolism's role in recovery (Thomas et al., 2022; Orešič et al., 2016). Elevated propionate and methionine in unfavorable outcomes further reflect altered energy metabolism and inflammation, while lower creatinine points to systemic effects (Sergeant et al., 2016; Yoshimura et al., 2021; Gu et al., 2016). Tryptophan metabolism's consistency across time points reinforces its role in driving neurotoxic versus neuroprotective kynurenine derivatives in ABI (Brouns et al., 2010).

Detailed analysis of individual metabolites, including their biological roles and specific associations with ABI outcomes, can be found in the supplemental discussion **(Supplement)**.

Although our study focused on serum, previous work has shown that metabolomic alterations in cerebrospinal fluid (CSF) also correlate with outcome after ABI. Certain CSF findings align with our results and enhance the mechanistic links between of metabolites — particularly amino acid and energy-related metabolites such as tryptophan and alanine (Lu et al., 2018). Experimental evidence from an ischemic stroke mouse model further supports early metabolic disruptions similar to those observed in our cohort, including changes in creatinine, lactate, L-alanine, and glutamic acid (Wang et al., 2013). Together, these studies suggest that serum metabolomics captures key metabolic responses also observed centrally, showing reflection of brain-specific processes. Future studies incorporating paired serum–CSF sampling will be important to clarify the degree of overlap and integrate peripheral and central metabolic signatures.

### 6.3 Prognostic implications and directions

The superior predictive power of our late model (AUC 94.4 %) compared to the early model (AUC 88.8 %) suggests that metabolomic signatures become more effective at distinguishing between outcome groups as secondary injury processes and recovery trajectories emerge. Combining early and late signatures (AUC 95.8 %) further boosts accuracy, indicating that longitudinal sampling could refine prognostic precision in clinical practice. Such an approach could guide early interventions (e.g., targeting energy metabolism or inflammation) while later profiles inform rehabilitation strategies. Our identification of novel biomarkers like PGJ2, GLA, and acetylated amino acids (NALA, NALS) opens avenues for mechanistic studies and therapeutic exploration. However, validation in larger, independent cohorts is essential to confirm these findings and address limitations such as sample size and treatment heterogeneity. Future studies should also explore integrating metabolomics with other omics data (e.g., proteomics, transcriptomics) to enhance predictive models, as demonstrated by Thomas et al.’s improved AUC with protein biomarkers (Thomas et al., 2022). In summary, our study advances the field by demonstrating that shared metabolomic signatures across ABI types can predict outcomes with high accuracy, with distinct early and late profiles reflecting the injury's temporal evolution.

## 7 Limitations

Our study has several limitations that should be considered when interpreting the findings. Firstly, the modest sample size of 73 participants constrains the generalizability and statistical power of our findings. Although we sought to enhance robustness by analyzing the serum metabolome at two distinct time points post-admission, replicating this study with a larger, multicentric cohort would improve statistical power and provide a more comprehensive understanding of metabolomic profiles across different populations and healthcare settings. Sample size expansion would facilitate the detection of more subtle metabolomic differences and enhance the applicability of these metabolic biomarkers for prognostic use in acute brain injury. Finally, the well-described cellular pathophysiological differences between traumatic brain injury and ischaemic stroke in lipidomic and metabolomic profiles (Thomas et al., 2022) may bias cross-etiology comparisons and further diminish effective statistical power.

Although our objective was to identify cross-injury commonalities, the absence of a comparator cohort, with healthy participants or patients with non-brain injuries, limits our ability to confirm which omic features are specific to acute brain injury. Inclusion of such comparators in future studies would help delineate ABI-specific signatures.

Additionally, certain variables, such as the administration of propofol and nutritional support, could potentially influence metabolomic changes in patients. Controlling for these factors was not feasible in this study. It is important to recognize that treatment protocols varied significantly across the types of ABI included in our study. For instance, patients with aSAH and aSDH were typically managed in the intensive care unit and often required prolonged sedation to manage complications like high intracranial pressure, hydrocephalus, or bleeding. Conversely, patients with IS were predominantly treated in the stroke unit, with most not requiring sedation. This variability in sedation and nutritional support across patient groups suggests that specific treatments, such as propofol, are unlikely to be the primary drivers of the significant metabolomic changes observed. Nonetheless, future studies could use randomization, stratified sampling, or propensity score matching to mitigate the influence of these confounding factors and strengthen the reliability of metabolomic associations.

Methodologically, the use of cross-validation within models such as PLS-DA and machine learning offers internal validation; however, it may not fully capture external variability, which can affect the generalizability of our findings. Our patient cohort and subcohorts are heterogeneous in diagnostic composition, and disease severity. This naturally limits statistical power and may compromise the reliability of our models. Validation of our findings with external identic cohorts would be necessary for the robustness and reproducibility of these metabolomic associations. Despite these limitations, our study leverages a rigorous methodological approach and a temporal analysis of metabolomic profiles, providing a new perspective on the evolving metabolic response to ABI. Temporal profiling allows us to capture active metabolic responses likely involved in recovery, supporting the relevance of the identified metabolites as potential prognostic biomarkers. These findings provide a first framework for future studies aimed at validating these biomarkers across diverse ABI types and clinical settings.

## 8 Conclusions

Research findings underscore the dynamic nature of metabolomic profiles in acute brain injuries and highlight metabolites as possible prognostic markers across various brain injury types. Longitudinally analyzed metabolomic changes may reflect underlying pathobiological processes, providing molecule candidates for further mechanistic validation. Further studies and validations of the identified metabolites in other cohorts and animal models are necessary to confirm these results and to elucidate the underlying pathobiological mechanisms linked to metabolism.

## Study approval and ethics

This study (T291/2016) was approved by the Institutional Review Board and Ethics Committee of Turku University Hospital. It adhered to the principles of the Declaration of Helsinki and its subsequent amendments. In cases where participants were unable to provide consent due to severe acute illness, written informed consent was obtained from their legal representatives. The study complied with all Finnish laws and regulations.

## Consent for publication

Not applicable.

## Availability of data and materials

The anonymized data from this study can be made available upon request to qualified researchers who have obtained appropriate institutional review board (IRB) approval. Requests should be directed to the corresponding author.

## Author contributions

The study was conceptualized, designed, and grant funded by J.K. Laboratory work was carried out by B.G., K.N., J.K., A.S., S.H., F.K., and S.K. Bioinformatic and statistical analyses were conducted by A.I.N. (bioinformatician), Y.C. (biostatistician), J.K., S.H., and A.S. Assessing patient outcomes was the responsibility of M.R., J.K., S.R., and F.K. The results were interpreted, and the initial manuscript was drafted by S.H., A.S., A.J., and J.K. S.H., JK and A.S. drafted the figures. The manuscript was critically reviewed, edited, and revised by J.F., T.R., S.B.L., Jo.F., R.T., J.P.P., S.R., F.K., M.J., S.P., A.A., A. Sr., R.G., A.I.N., M.R., J.R., and E.C. All authors have read and approved the final version of the manuscript for submission.

## Funding

Funding for this work was provided to J.K. by the Sigrid Juselius Foundation and the 10.13039/100008723Finnish Medical Foundation. A.S. received support from both the Sigrid Juselius Foundation and the Maire Taponen Foundation, while S.H. was funded by the 10.13039/501100006306Sigrid Jusélius Foundation. J.P.P. is supported by the Research Council of Finland and Sigrid Jusélius Foundation, and State Research Funding of Finland.

## Declaration of competing interest

The authors declare the following financial interests/personal relationships which may be considered as potential competing interests:Santtu Hellstrom reports financial support was provided by Sigrid Jusélius Foundation. Janne Koskimaki reports financial support was provided by Sigrid Jusélius Foundation. Janne Koskimaki reports financial support was provided by Finnish Medical Foundation. Antti Sajanti reports financial support was provided by Maire Taponen Foundation. Jussi P. Posti reports financial support was provided by Sigrid Jusélius Foundation. Jussi P. Posti reports financial support was provided by Research Council of Finland. Jussi P. Posti reports financial support was provided by State research council Finland. If there are other authors, they declare that they have no known competing financial interests or personal relationships that could have appeared to influence the work reported in this paper.

## List of abbreviations

ABI: Acute Brain Injury
aSAH: Aneurysmal Subarachnoid Hemorrhage
IS: Ischemic Stroke
TBI: Traumatic Brain Injury
AD: Alzheimer's Disease
PD: Parkinson's Disease
HD: Huntington's Disease
ALS: Amyotrophic Lateral Sclerosis
MS: Multiple Sclerosis
AA: Amino Acid
PE: Phosphatidylethanolamine
PC: Phosphatidylcholine
LysoPE: Lysophosphatidylethanolamine
LysoPC: Lysophosphatidylcholine
EPC: Etherphosphatidylcholine
PLA2: Phospholipase A2
PLAC: Phospholipase C
AUC: Area Under the Curve
FA: Fatty Acid
BCAA: Branched-chain Amino Acid
mRS: Modified Ranking Scale
QC: Quality Control
PLS-DA: Partial Least Squares Discriminant Analysis
SVM: Support Vector Machine
LDA: Linear Discriminant Analysis
ROC: Receiver Operating Characteristic
KEGG: Kyoto Encyclopedia of Genes and Genomes
QEA: Quantitative Enrichment Analysis
FDR: False Discovery Rate
GCS: Glasgow Coma Scale
KM: K-Means
PGJ2: Prostaglandin J2
GLA: Gamma-Linolenic Acid

## References

[bib1] Ahmed W., White I.R., Wilkinson M., Johnson C.F., Rattray N., Kishore A.K. (2021). Breath and plasma metabolomics to assess inflammation in acute stroke. Sci. Rep..

[bib2] Banoei M.M., Lee C.H., Hutchison J., Panenka W., Wellington C., Wishart D.S. (2023). Using metabolomics to predict severe traumatic brain injury outcome (GOSE) at 3 and 12 months. Crit Care Lond Engl.

[bib3] Benjamini Y., Hochberg Y. (1995). Controlling the false discovery rate: a practical and powerful approach to multiple testing. J R Stat Soc Ser B Methodol.

[bib4] Brouns R., Verkerk R., Aerts T., De Surgeloose D., Wauters A., Scharpé S. (2010). The role of tryptophan catabolism along the kynurenine pathway in acute ischemic stroke. Neurochem. Res..

[bib5] Capizzi A., Woo J., Verduzco-Gutierrez M. (2020). Traumatic brain injury: an overview of epidemiology, pathophysiology, and medical management. Med. Clin..

[bib6] Carney N., Totten A.M., O'Reilly C., Ullman J.S., Hawryluk G.W.J., Bell M.J. (2017). Guidelines for the management of severe traumatic brain injury. Neurosurgery.

[bib7] Chi N.F., Chang T.H., Lee C.Y., Wu Y.W., Shen T.A., Chan L. (2021). Untargeted metabolomics predicts the functional outcome of ischemic stroke. J. Formos. Med. Assoc..

[bib8] Connolly E.S., Rabinstein A.A., Carhuapoma J.R., Derdeyn C.P., Dion J., Higashida R.T. (2012). Guidelines for the management of aneurysmal subarachnoid hemorrhage: a guideline for healthcare professionals from the American heart association/American stroke association. Stroke.

[bib9] Dagonnier M., Donnan G.A., Davis S.M., Dewey H.M., Howells D.W. (2021). Acute stroke biomarkers: are we there yet?. Front. Neurol..

[bib10] Dale N., Tian F., Sagoo R., Phillips N., Imray C., Roffe C. (2019). Point-of-care measurements reveal release of purines into venous blood of stroke patients. Purinergic Signal..

[bib11] Dempsey R.J., Raghavendra Rao V.L. (2003). Cytidinediphosphocholine treatment to decrease traumatic brain injury-induced hippocampal neuronal death, cortical contusion volume, and neurological dysfunction in rats. J. Neurosurg..

[bib12] Dijkland S., Donkelaar K.V., Van den Bergh W., Bakker J., Dippel D., Nijsten M. (2015). Prognostic value of blood lactate and glucose levels after aneurysmal subarachnoid hemorrhage. Crit. Care.

[bib13] Feigin V.L., Brainin M., Norrving B., Martins S., Sacco R.L., Hacke W. (2022). World stroke organization (WSO): global stroke fact sheet 2022. Int J Stroke Off J Int Stroke Soc.

[bib14] Girard R., Zeineddine H.A., Koskimäki J., Fam M.D., Cao Y., Shi C. (2018). Plasma biomarkers of inflammation and angiogenesis predict cerebral cavernous malformation symptomatic hemorrhage or lesional growth. Circ. Res..

[bib15] Goeman J.J., van de Geer S.A., de Kort F., van Houwelingen H.C. (2004). A global test for groups of genes: testing association with a clinical outcome. Bioinforma Oxf Engl.

[bib16] Gold A.B., Herrmann N., Swardfager W., Black S.E., Aviv R.I., Tennen G. (2011). The relationship between indoleamine 2,3-dioxygenase activity and post-stroke cognitive impairment. J. Neuroinflammation.

[bib17] Goutman S.A., Boss J., Guo K., Alakwaa F.M., Patterson A., Kim S. (2020). Untargeted metabolomics yields insight into ALS disease mechanisms. J. Neurol. Neurosurg. Psychiatry.

[bib18] Gu S.X., Blokhin I.O., Wilson K.M., Dhanesha N., Doddapattar P., Grumbach I.M. (2016). Protein methionine oxidation augments reperfusion injury in acute ischemic stroke. JCI Insight [Internet].

[bib19] Hajsl M., Hlavackova A., Broulikova K., Sramek M., Maly M., Dyr J.E. (2020). Tryptophan metabolism, inflammation, and oxidative stress in patients with neurovascular disease. Metabolites.

[bib20] Hellström S., Sajanti A., Srinath A., Bennett C., Girard R., Jhaveri A. (2025). Common lipidomic signatures across distinct acute brain injuries in patient outcome prediction. Neurobiol. Dis..

[bib21] Huo Z., Yu L., Yang J., Zhu Y., Bennett D.A., Zhao J. (2020). Brain and blood metabolome for Alzheimer's dementia: findings from a targeted metabolomics analysis. Neurobiol. Aging.

[bib22] Hussain G., Anwar H., Rasul A., Imran A., Qasim M., Zafar S. (2020). Lipids as biomarkers of brain disorders. Crit. Rev. Food Sci. Nutr..

[bib23] Jeter C.B., Hergenroeder G.W., Ward N.H., Moore A.N., Dash P.K. (2013). Human mild traumatic brain injury decreases circulating branched-chain amino acids and their metabolite levels. J. Neurotrauma.

[bib24] Jung J.Y., Lee H.S., Kang D.G., Kim N.S., Cha M.H., Bang O.S. (2011). 1H-NMR-Based metabolomics study of cerebral infarction. Stroke.

[bib25] Kabadi S.V., Maher T.J. (2010). Posttreatment with uridine and melatonin following traumatic brain injury reduces edema in various brain regions in rats. Ann. N. Y. Acad. Sci..

[bib26] Kanehisa M., Goto S. (2000). KEGG: kyoto encyclopedia of genes and genomes. Nucleic Acids Res..

[bib27] Liu H., Li W., Ahmad M., Rose M.E., Miller T.M., Yu M. (2013). Increased generation of cyclopentenone prostaglandins after brain ischemia and their role in aggregation of ubiquitinated proteins in neurons. Neurotox. Res..

[bib28] Liu M., Zhou K., Li H., Dong X., Tan G., Chai Y. (2015). Potential of serum metabolites for diagnosing post-stroke cognitive impairment. Mol. Biosyst..

[bib29] Liu Z., Waters J., Rui B. (2022). Metabolomics as a promising tool for improving understanding of multiple sclerosis: a review of recent advances. Biomed. J..

[bib30] Lu A.Y., Damisah E.C., Winkler E.A., Grant R.A., Eid T., Bulsara K.R. (2018). Cerebrospinal fluid untargeted metabolomic profiling of aneurysmal subarachnoid hemorrhage: an exploratory study. Br. J. Neurosurg..

[bib31] Maas A.I.R., Menon D.K., Manley G.T., Abrams M., Åkerlund C., Andelic N. (2022). Traumatic brain injury: progress and challenges in prevention, clinical care, and research. Lancet Neurol..

[bib32] Mastrokolias A., Pool R., Mina E., Hettne K.M., van Duijn E., van der Mast R.C. (2016). Integration of targeted metabolomics and transcriptomics identifies deregulation of phosphatidylcholine metabolism in Huntington's disease peripheral blood samples. Metabolomics.

[bib33] Mo X., Pi L., Yang J., Xiang Z., Tang A. (2014). Serum indoleamine 2,3-dioxygenase and kynurenine aminotransferase enzyme activity in patients with ischemic stroke. J. Clin. Neurosci..

[bib34] Oft H.C., Simon D.W., Sun D. (2024). New insights into metabolism dysregulation after TBI. J. Neuroinflammation.

[bib35] Orešič M., Posti J.P., Kamstrup-Nielsen M.H., Takala R.S.K., Lingsma H.F., Mattila I. (2016). Human serum metabolites associate with severity and patient outcomes in traumatic brain injury. EBioMedicine.

[bib36] Pang Z., Chong J., Zhou G., de Lima Morais D.A., Chang L., Barrette M. (2021). MetaboAnalyst 5.0: narrowing the gap between raw spectra and functional insights. Nucleic Acids Res..

[bib37] Powers W.J., Rabinstein A.A., Ackerson T., Adeoye O.M., Bambakidis N.C., Becker K. (2019). Guidelines for the early management of patients with acute ischemic stroke: 2019 update to the 2018 guidelines for the early management of acute ischemic stroke: a guideline for healthcare professionals from the American heart association/American stroke association. Stroke.

[bib38] Ricciotti E., FitzGerald G.A. (2011). Prostaglandins and inflammation. Arterioscler. Thromb. Vasc. Biol..

[bib39] Sajanti A., Hellström S., Bennett C., Srinath A., Jhaveri A., Cao Y. (2024). Soluble urokinase-type plasminogen activator receptor and inflammatory biomarker response with prognostic significance after acute neuronal injury – a prospective cohort study. Inflammation.

[bib40] Sergeant S., Rahbar E., Chilton F.H. (2016). Gamma-linolenic acid, Dihommo-gamma linolenic, Eicosanoids and inflammatory processes. Eur. J. Pharmacol..

[bib41] Shao Y., Li T., Liu Z., Wang X., Xu X., Li S. (2021). Comprehensive metabolic profiling of Parkinson's disease by liquid chromatography-mass spectrometry. Mol. Neurodegener..

[bib42] Shin T.H., Lee D.Y., Basith S., Manavalan B., Paik M.J., Rybinnik I. (2020). Metabolome changes in cerebral ischemia. Cells.

[bib43] Sidorov E., Sanghera D.K., Vanamala J.K.P. (2019). Biomarker for ischemic stroke using metabolome: a clinician perspective. J Stroke.

[bib44] Srinath A., Xie B., Li Y., Sone J.Y., Romanos S., Chen C. (2023). Plasma metabolites with mechanistic and clinical links to the neurovascular disease cavernous angioma. Commun. Med..

[bib45] Sun G., Jiang F., Hu S., Cheng H., Qu L., Tao Y. (2022). Metabolomic analysis reveals potential biomarkers and serum metabolomic profiling in spontaneous intracerebral hemorrhage patients using UPLC/quadrupole time-of-flight MS. Biomed. Chromatogr..

[bib46] Svedung Wettervik T., Engquist H., Howells T., Rostami E., Hillered L., Enblad P. (2020). Arterial lactate in traumatic brain injury - relation to intracranial pressure dynamics, cerebral energy metabolism and clinical outcome. J. Crit. Care.

[bib47] Tao S., Xiao X., Li X., Na F., Na G., Wang S. (2023). Targeted metabolomics reveals serum changes of amino acids in mild to moderate ischemic stroke and stroke mimics. Front. Neurol..

[bib48] Thomas I., Dickens A.M., Posti J.P., Czeiter E., Duberg D., Sinioja T. (2022). Serum metabolome associated with severity of acute traumatic brain injury. Nat. Commun..

[bib49] Tibshirani R. (1996). Regression shrinkage and selection via the lasso. J R Stat Soc Ser B Stat Methodol.

[bib50] Wang Y., Wang Y., Li M., Xu P., Gu T., Ma T. (2013). (1)H NMR-based metabolomics exploring biomarkers in rat cerebrospinal fluid after cerebral ischemia/reperfusion. Mol. Biosyst..

[bib51] Wieloch T., Siesjö B.K. (1982). Ischemic brain injury: the importance of calcium, lipolytic activities, and free fatty acids. Pathol. Biol..

[bib52] Xia J., Psychogios N., Young N., Wishart D.S. (2009). MetaboAnalyst: a web server for metabolomic data analysis and interpretation. Nucleic Acids Res..

[bib53] Yan E.B., Frugier T., Lim C.K., Heng B., Sundaram G., Tan M. (2015). Activation of the kynurenine pathway and increased production of the excitotoxin quinolinic acid following traumatic brain injury in humans. J. Neuroinflammation.

[bib54] Yang L., Lv P., Ai W., Li L., Shen S., Nie H. (2017). Lipidomic analysis of plasma in patients with lacunar infarction using normal-phase/reversed-phase two-dimensional liquid chromatography–quadrupole time-of-flight mass spectrometry. Anal. Bioanal. Chem..

[bib55] Yoshimura Y., Wakabayashi H., Nagano F., Bise T., Shimazu S., Shiraishi A. (2021). Elevated creatinine-based estimated glomerular filtration rate is associated with increased risk of Sarcopenia, Dysphagia, and reduced functional recovery after stroke. J. Stroke Cerebrovasc. Dis..

[bib56] Youden W.J. (1950). Index for rating diagnostic tests. Cancer.

[bib57] Zhang X., Lu X., Shi Q., Xu X.Q., Leung H.C., Harris L.N. (2006). Recursive SVM feature selection and sample classification for mass-spectrometry and microarray data. BMC Bioinf..

[bib58] Zhao X., Zhang Y., Strong R., Grotta J.C., Aronowski J. (2006). 15d-Prostaglandin J2 activates peroxisome proliferator-activated Receptor-γ, promotes expression of catalase, and reduces inflammation, behavioral dysfunction, and neuronal loss after intracerebral hemorrhage in rats. J. Cerebr. Blood Flow Metabol..

