# Supplementary material for: Shared metabolomic signatures for prognostic precision across brain injuries
Source: Brain Spine. 2025 Nov 19;5:105877. doi: 10.1016/j.bas.2025.105877 (PMC12682153; doi:10.1016/j.bas.2025.105877)
Supplement: Multimedia component 1 [file mmc1.docx]

| **Supplemental Table S1. Demographic and disease characteristics of the study cohort. General information, N=73.** | | | |
| --- | --- | --- | --- |
| **Variable** | **Features** | **N = 73** | **%** |
| Gender | Male | 40 | 54.8 |
|  | Female | 33 | 45.2 |
|  | | | |
| General Health | Excellent = no diseases | 14 | 19.2 |
|  | Good = mild diseases, normal health | 32 | 43.8 |
|  | Moderate = some significant diseases which affects general health | 25 | 34.3 |
|  | Poor = unable to work because of general health | 2 | 2.9 |
|  | | | |
| Earlier brain injuries | No | 64 | 88.7 |
|  | Yes | 9 | 12.3 |
|  | | | |
| Alcohol abuse | No | 53 | 72.6 |
|  | Yes | 20 | 27.4 |
|  | | | |
| Drug abuse | No | 71 | 97.3 |
|  | Yes | 2 | 2.7 |
|  |  |  |  |
|  | | | |
| Smoking | No | 42 | 57.5 |
|  | Yes | 31 | 42.5 |
|  | | | |
| Type of brain injury | aSAH | 30 | 41.1 |
|  | TBI | 13 | 17.8 |
|  | IS | 30 | 41.1 |
|  | | | |
| Location of injury | Frontal lobes | 15 | 20.5 |
|  | Parietal lobes | 2 | 2.7 |
|  | Temporal lobes | 34 | 46.6 |
|  | Occipital lobes | 1 | 1.4 |
|  | Brain stem | 3 | 4.1 |
|  | Cerebellum | 9 | 12.3 |
|  | | | |
| Location of infarction | MCA | 20 | 27.4 |
|  | ACA | 5 | 6.8 |
|  | PCA | 5 | 6.8 |
|  | Basilar | 2 | 2.7 |
|  | Lacunar | 4 | 5.5 |
|  | Vertebral | 2 | 2.7 |
|  | No Infarction | 35 | 48.6 |
|  | | | |
| Basal cisterns compression | No | 39 | 53.4 |
|  | Absent | 8 | 11.0 |
|  | Compressed | 26 | 35.6 |
|  | | | |
| IVH component | No | 46 | 63.0 |
|  | Yes | 27 | 37.0 |
|  | | | |
| ICH component | No | 42 | 57.5 |
|  | Yes | 31 | 42.5 |
|  | | | |
| Hemorrhagic infarction | No | 66 | 90.4 |
|  | Yes | 7 | 9.6 |
|  | | | |
| Neuroworsening | No | 38 | 52.1 |
|  | Yes | 35 | 47.9 |
|  | | | |
| Alcohol involved  Missing values N=5 | No | 63 | 92.6 |
|  | Yes | 5 | 7.4 |
|  | | | |
| ICP monitoring | No | 44 | 59.4 |
|  | Yes | 29 | 40.6 |
|  | | | |
| Hemicraniectomy | No | 68 | 92.8 |
|  | Yes | 5 | 7.2 |
|  | | | |
| External ventricular drainage | No | 47 | 62.9 |
|  | yes | 26 | 37.1 |
|  | | | |
| Infection | No | 49 | 67.1 |
|  | Yes | 24 | 32.9 |
|  | | | |
| Diagnosis of acute hydrocephalus | No | 58 | 79.5 |
|  | Yes | 15 | 20.5 |
|  | | | |
| Diagnosis of shunt dependent hydrocephalus  Missing values, N=1 | No | 60 | 83.3 |
|  | Yes | 12 | 16.7 |
|  | | | |
| Neuropsychological symptoms  Missing values, N=12 | No | 27 | 44.1 |
|  | Yes | 34 | 55.9 |
|  |  |  |  |
| GCS scene | Mean | 11.92 | - |
|  | Median | 15 | - |
|  | Minimum | 3 | - |
|  | Maximum | 15 | - |
|  | Std Dev | 4.33 | - |
|  |  |  |  |
| Length of ICU treatment period (days) | Mean | 6.68 | - |
|  | Median | 3 | - |
|  | Minimum | 0 | - |
|  | Maximum | 53 | - |
|  | Std Dev | 10.16 | - |
|  | | | |
| **Supplemental Table S1. Demographic and disease characteristics of the study cohort. Ischemic stroke (IS), N=30.** | | | |
| **Variable** | **Features** | **N = 30** | **%** |
| Type of infarct | Cardiogenic | 7 | 23.3 |
|  | Thrombotic | 10 | 33.3 |
|  | Cryptogenic | 13 | 43.4 |
|  | | | |
| TOAST classification  Missing values, N=5 | 1 | 5 | 23.3 |
|  | 2 | 6 | 24.0 |
|  | 3 | 1 | 4.0 |
|  | 4 | 2 | 8.0 |
|  | 5 | 12 | 48.0 |
|  | | | |
| NIHSS | Mean | 7.429 | - |
|  | Median | 6 | - |
|  | Minimum | 0 | - |
|  | Maximum | 20 | - |
|  | Std Dev | 6.30 | - |
|  |  |  |  |
| Volume of infarction | Mean | 47.63 | - |
|  | Median | 24.59 | - |
|  | Minimum | 0.10 | - |
|  | Maximum | 303 | - |
|  | Std Dev | 71.27 | - |
|  | | | |
| **Supplemental Table S1. Demographic and disease characteristics of the study cohort. Aneurysmal subarachnoid hemorrhage (aSAH), N=30.** | | | |
| **Variable** | **Features** | **N = 30** | **%** |
| modified Fisher Scale | 1 | 1 | 3.3 |
|  | 2 | 3 | 10.0 |
|  | 3 | 4 | 13.3 |
|  | 4 | 22 | 73.3 |
|  | | | |
| Hunt & Hess | 1 | 10 | 33.3 |
|  | 2 | 6 | 20.0 |
|  | 3 | 4 | 13.3 |
|  | 4 | 5 | 16.7 |
|  | 5 | 5 | 16.7 |
|  | | | |
| WFNS | 1 | 10 | 33.3 |
|  | 2 | 8 | 26.7 |
|  | 3 | 2 | 6.7 |
|  | 4 | 2 | 6.7 |
|  | 5 | 8 | 26.7 |
|  | | | |
| Clinical DCI | No | 20 | 66.7 |
|  | Yes | 10 | 33.3 |
|  | | | |
| Location of Aneurysm | Anterior circulation | 27 | 90.0 |
|  | Posterior circulation | 3 | 10.0 |
|  | | | |
| Location of Aneurysm | Anterior circulation | 27 | 90.0 |
|  | Posterior circulation | 3 | 10.0 |
|  | | | |
| Location of Aneurysm specific | ACA | 2 | 6.7 |
|  | AcomA | 10 | 33.3 |
|  | MCA | 5 | 16.7 |
|  | PICA | 2 | 6.7 |
|  | AICA | 3 | 10.0 |
|  | SCA | 1 | 3.3 |
|  | ICA | 7 | 23.3 |
|  | | | |
| Aneurysm Deformed | No | 13 | 43.3 |
|  | Yes | 17 | 56.7 |
|  | | | |
| Type of Aneurysm | Saccular | 19 | 63.3 |
|  | Fusiform | 11 | 36.7 |
|  | | | |
| Size of aneurysm max length (mm) | Mean | 6.74 | - |
|  | Median | 5.10 | - |
|  | Minimum | 1.30 | - |
|  | Maximum | 20 | - |
|  | Std Dev | 5.00 | - |
|  |  |  |  |
| Size of aneurysm max width (mm) | Mean | 5.73 | - |
|  | Median | 3.65 | - |
|  | Minimum | 1.50 | - |
|  | Maximum | 17 | - |
|  | Std Dev | 4.45 | - |
|  | | | |
| Width of aneurysm neck (mm) | Mean | 2.88 | - |
|  | Median | 2.85 | - |
|  | Minimum | 1.30 | - |
|  | Maximum | 8.55 | - |
|  | Std Dev | 1.35 | - |
|  | | | |
| **Supplemental Table S1. Demographic and disease characteristics of the study cohort. TBI (aSDH), N=13** | | | |
| **Variable** | **Features** | **N = 13** | **%** |
| aSDH location | Right | 7 | 53.8 |
|  | Left | 6 | 42.2 |
|  | | | |
| Midline shift (mm) | Mean | 2.61 | - |
|  | Median | 0 | - |
|  | Minimum | 0 | - |
|  | Maximum | 20 | - |
|  | Std Dev | 4.59 | - |
|  | | | |
| Volume of aSDH (ml) | Mean | 56.26 | - |
|  | Median | 46.0 | - |
|  | Minimum | 0.40 | - |
|  | Maximum | 122 | - |
|  | Std Dev | 45.85 | - |
|  | | | |
| aSDH thicness (mm) | Mean | 12.12 | - |
|  | Median | 12.0 | - |
|  | Minimum | 4.50 | - |
|  | Maximum | 20 | - |
|  | Std Dev | 6.09 | - |
|  | | | |
