# Supplementary material for: Shared metabolomic signatures for prognostic precision across brain injuries
Source: Brain Spine. 2025 Nov 19;5:105877. doi: 10.1016/j.bas.2025.105877 (PMC12682153; doi:10.1016/j.bas.2025.105877)
Supplement: Multimedia component 5 [file mmc5.docx]

**Shared metabolomic signatures for prognostic precision across brain injuries**

Santtu Hellström MD^1,2^, Antti Sajanti MD^1,2^, Aditya Jhaveri BS^3^, Ying Cao MS^4^, Fredrika Koskimäki MD^5^, Johannes Falter MD^6^, Janek Frantzén MD, PhD^1,2^, Seán B. Lyne MD^7^, Tomi Rantamäki PhD^8, 9^, Riikka Takala MD, PhD^10^, Jussi P. Posti MD, PhD^1,2^, Susanna Roine MD, PhD^4^, Sulo Kolehmainen BS^11^, Bharat Gajera^12^, Kenneth Nazir^12^, Miro Jänkälä MD^13^, Susanna Piironen MD^13^, Ahmed Abdirisak MD, PhD^13^, Abhinav Srinath PhD^3^, Romuald Girard PhD^3^, Anni I Nieminen PhD^12^, Melissa Rahi MD, PhD^1,2^, Jaakko Rinne MD, PhD^1,2^, Eero Castrén MD, PhD^11^, Janne Koskimäki MD, PhD^1, 2, 11, 13^

**Author affiliations:**

^1^Neurocenter, Department of Neurosurgery, Turku University Hospital and University of Turku, P.O. Box 52, FI-20521, Turku, Finland.

^2^Division of Clinical Neurosciences, Neurosurgery, University of Turku, P.O. Box 52, FI-20521, Turku, Finland.

^3^Neurovascular Surgery Program, Section of Neurosurgery, The University of Chicago Medicine and Biological Sciences, Chicago, IL 60637, US.

^4^Department of Radiation Oncology, Kansas University Medical Center, Kansas City, KS 66160, USA.

^5^Neurocenter, Acute Stroke Unit, Turku University Hospital, P.O. Box 52, FI-20521, Turku, Finland.

^6^Department of Neurosurgery, University Medical Center of Regensburg, Regensburg, 93042, Germany.

^7^Department of Neurosurgery, Brigham and Women’s Hospital, Harvard Medical School, Boston, MA, US.

^8^Laboratory of Neurotherapeutics, Drug Research Program, Division of Pharmacology and Pharmacotherapy, Faculty of Pharmacy, University of Helsinki, P.O. Box 56, FI-00014 Helsinki, Finland.

^9^SleepWell Research Program, Faculty of Medicine, University of Helsinki, P.O. Box 63, FI-00014 Helsinki, Finland.

^10^Perioperative Services, Intensive Care and Pain Medicine and Department of Anaesthesiology and Intensive Care, Turku University Hospital and University of Turku, P.O. Box52, FI-20521, Turku, Finland.

^11^Neuroscience Center, HiLIFE, University of Helsinki, P.O. Box 63, FI-00014 Helsinki, Finland.

^12^Helsinki Metabolomics Center, Faculty of Medicine, University of Helsinki, Finland.

^13^Department of Neurosurgery, Oulu University Hospital, Box 25, 90029 OYS, Finland.

**Correspondence to**: Janne Koskimäki, MD, PhD, Associate Professor

**Full address**: Neurocenter, Department of Neurosurgery, Turku University Hospital and University of Turku, P.O. Box 52 (Hämeentie 11), FI-20521, Turku, Finland.

**E-mail:** jankosk@utu.fi

**Running head:** Acute Brain Injury Unified Metabolomic Insights

**Keywords:** brain injury; metabolomics; outcome; prognosis; stroke; traumatic brain injury

**Supplementary materials and methods:**

**Targeted liquid chromatography-mass spectrometry (LC-MS) metabolomics profiling analytics**

Metabolites were extracted from 100µl human serum with 400µl of cold extraction solvent (Acetonitrile:Methanol:MQ; 40:40:20, Thermo Fisher Scientific). Subsequently, samples were vortexed for 2 minutes and sonicated for 1 minute followed by centrifugation at 14000rpm at 4°C for 5 minutes. Supernatants were transferred into polypropylene tubes and placed into a Nitrogen gas evaporator. Dried samples were reconstituted with 40µl of extraction solvent (ACN:MeOH:MQ; 40:40:20, Thermo Fisher Scientific), vortexed for 2 minutes, and transferred into HPLC glass autosampler vials. Finally, 2µl of samples were injected into Thermo Vanquish UHPLC coupled with Q-Exactive Orbitrap MS set to a MS1/full scanning (55-825 m/z) mode and equipped with a heated electrospray ionization (H-ESI) source probe (Thermo Fisher Scientific). A SeQuant ZIC-pHILIC (2.1×100 mm, 5-μm particle) column (Merck) was used for chromatographic separation, flow rate of 0.100 mL/minutes, gradient in 24 min (from 80%B to 20%), 20mM Ammonium hydrogen Carbonate, pH 9.4 as mobile phase solutions A and Acetonitrile as B. Instrument was operated with the Xcalibur 4.1.31.9 software (Thermo Fisher Scientific), and the peak integration was performed with the TraceFinder 4.1 SP2 software (Thermo Fisher Scientific) for 136 metabolites standardized with library kit MSMLS-1EA (Merck). The data quality was monitored throughout the run using both a pooled Quality Control (QC) prepared by pooling 5µl from each study sample and an inhouse control serum sample interspersed throughout the run as every 10th sample.

**Supplementary results:**

**Demographics and disease characteristics of enrolled patients**

A breakdown of our cohort according to disease groups is presented in Table S1, detailing specific clinical characteristics associated with the acute brain injury (ABI) cohort. A slightly higher proportion of patients were men (40/73, 54.8%). The patient cohort was divided into disease-specific subgroups including aneurysmal subarachnoid hemorrhage, ischemic stroke, and traumatic brain injury (aSAH, IS, and TBI respectively) with 30 (41.1%), 30 (41.10%), and 13 (17.8%) patients, respectively (**Table S1**) Eight (11.4%) patients in the study cohort had a history of previous brain injury. In the study cohort, 20 (27.4%) reported alcohol abuse, with alcohol involvement in 5 (7.4%) ABI cases. Additionally, 31 (42.5%) patients were smokers. Drug abuse was reported with 2 (2.7%) patients. In addition, we gathered data from disease-specific subgroups for a more focused examination of key variables within these subsets.

**Ischemic stroke**

In the IS subcohort, we recorded the type of infarction and infarction volume among others (**Table S1**). The distribution of infarctions varied among six different arteries, with the most prevalent location identified as the middle cerebral artery (MCA), affecting 20 patients. Infarct etiology was classified as cryptogenic in 13 (43.4%), thrombotic in 10 (33.3%), and cardiogenic in 7 (23.3%), reflecting a predominance of undetermined causes. Infarction volumes exhibited a range from 0.10 to 303 mL, with a mean volume of 47.63 mL and a standard deviation (SD) of 71.27 mL (**Table S1**). Stroke severity, assessed via the National Institutes of Health Stroke Scale (NIHSS) at presentation, had a mean score of 7.43 ± 6.30, a median of 6, and ranged from 0 to 20 **(Table S1)**.

**Aneurysmal subarachnoid hemorrhage**

In the aSAH cohort, the predominant location of aneurysms was in the anterior circulation, accounting for 27 out of 30 cases (90%) (**Table S1**). Notably, the most frequently observed individual location was the anterior communicating artery (AcomA), accounting for 10 cases (33.3%). The second most prevalent location was the internal carotid artery (ICA), identified in 7 of the cases (23.3%) (**Table S1**). Hunt and Hess (H&H) scores ranged from 1 to 5, demonstrating a relatively even distribution: 10 cases (33.3%) scored 1, 6 cases (20.0%) scored 2, 4 cases (13.3%) scored 3, and 5 cases each (16.7%) scored 4 and 5, respectively (**Table S1**). Aneurysms were classified as saccular in 63.3% (19/30) and fusiform in 36.7% (11/30). Maximum aneurysm length averaged 6.74 ± 5.00 mm (median 5.10 mm, range 1.30–20 mm), while maximum width averaged 5.73 ± 4.45 mm (median 3.65 mm, range 1.50–17 mm). Both dimensions showed right-skewed distributions, with most aneurysms small-to-medium but a few reaching larger sizes (e.g., 20 mm length, 17 mm width).

**Traumatic brain injury**

We completed further analysis for the traumatic brain injury (TBI) subcohort and measured the thickness of acute subdural hematoma (aSDH) and midline shift. The mean thickness of aSDH was 12.12 ± 6.09 mm, with a median of 12.0 mm. The largest observed aSDH measured 20 mm. The mean midline shift measured 2.61 ± 4.59 mm (**Table S1**). The locations were divided between the right and left hemispheres, with 7 cases (53.8%) in the right hemisphere and 6 cases (42.2%) in the left hemisphere (**Table S1**).

**Metabolomic PCA reveals no distinct clustering across brain injury groups**

The principal component analysis (PCA) of metabolomic profiles across different brain injury groups was conducted to assess metabolomic signatures associated with specific clinical outcomes at distinct time points (**Supplemental Figure S1**). The analysis included four panels representing early favorable (A), early unfavorable (B), late favorable (C), and late unfavorable (D) outcomes. Across all panels, PCA failed to demonstrate distinct clustering by disease group, suggesting a lack of unique metabolomic signatures differentiating aSAH, IS, and TBI within the same outcome category.

**Supplemental Figure S1. Principal component analysis (PCA) of lipidomic profiles across different brain Injury groups with specific outcomes**. Each panel represents a different clinical outcome observed at distinct time points: early favorable (**A**), early unfavorable (**B**), late favorable (**C**), and late unfavorable (**D**). **A-D**) In all four panels, PCA did not reveal distinct clustering by disease group, indicating a lack of distinguishable metabolomic signatures between aneurysmal subarachnoid hemorrhage (aSAH), ischemic stroke (IS), and traumatic brain injury (TBI) within the same outcome category.

**Linear discriminant model combining early and late metabolites robustly predicts outcome**

A linear discriminant analysis (LDA) model was constructed using selected early and late candidate metabolites to predict favorable outcomes. The model showed excellent performance with an AUC of 95.8% (95% CI 91–100%), OR 12.5 (95% CI 3.27–47.6), sensitivity of 87%, and specificity of 97% (Youden index J = 0.84, p = 0.0002). The derived canonical equation integrates information across time points, highlighting the added value of combining early and late markers (**Supplemental Figure S2**).

**Supplemental Figure S2. Linear discriminant analyses (LDA) of the selected candidate biomarkers combining early and late canonical predictive information.** The receiver operating characteristic curve of selected candidate metabolites in after combinatory LDA prognosing favorable outcome: odds ratio (OR) 12.5 (95% confidence interval (CI) 3.27–47.6); Area under the curve (AUC) = 95.8%, 95% CI = (91–100%), p = 0.0002, with 87% sensitivity and 97% specificity J = 0.84). LDA of these metabolites resulted in an equation with canonical scores: 0.240[Uridine early] + 0.211[Tryptophan early] – 0.216[Lactic acid early] + 0.266[Prostaglandin J2 late] – 0.544[Gamma-linolenic acid late] – 0.003[N-acetyl-L-alanine late] + 0.175[Uridine late] – 0.174[N-alpha-acetyl-L-asparagine late] – 0.320[3-hydroxy-3-methylglutarate late] – 0.287[Propionate late] + 0.573[Creatinine late]. Optimal cut-off point for the prognostic test was determined by calculating the Youden index (J).

**Supplementary discussion**

**Breaking down the metabolite models**

**Metabolites of early time point**

In blood samples taken during the early phase, the biomarkers that demonstrated the strongest prognostic value were uridine, tryptophan, and lactic acid. Based on these three biomarkers, we developed a prognostic model that achieved a robust AUC of 88.8%.

**Uridine**stood out as a significant predictor of outcomes in both early and late sample groups, with the strongest prognostic value in the early phase. In the central nervous system, uridine supports memory and neuroplasticity (1). Its effects are likely related to its involvement in membrane formation, a process in which it participates as an intermediator in the Kennedy cycle (2). Preclinical studies in mice demonstrate that uridine and its metabolite CDP-choline reduce cerebral edema, brain damage, and neuronal loss following injury (3,4). In our cohort, lower uridine levels correlated with unfavorable outcomes (mRS 4-6), possibly reflecting increased membrane repair demands in severe ABI that deplete circulating uridine. This could account for its further reduction in late samples. Alternatively, lower baseline uridine levels may impair neuroprotection, contributing to poorer recovery, though this requires further investigation.

**Tryptophan**, an essential amino acid, is metabolized primarily via two pathways: the kynurenine pathway (KP), accounting for approximately 95% of its catabolism, and the serotonin pathway, comprising about 5% (5). The KP influences diverse functions, notably inflammation and immune responses. Within this pathway, tryptophan is converted to kynurenine, which branches into either neuroprotective kynurenic acid or neurotoxic quinolinic acid. Following ABI, inflammation shifts KP metabolism toward the neurotoxic branch, enhancing quinolinic acid production (6). Clinical studies report lower tryptophan levels in IS patients compared to healthy controls (7), alongside elevated kynurenine-to-tryptophan (KYN/TRP) ratios in those with poorer outcomes, suggesting KP activity correlates with stroke severity and prognosis (8). These observations align with our findings, where patients with unfavorable outcomes exhibited reduced tryptophan levels relative to those with favorable outcomes. Moreover, tryptophan metabolism emerged as a significant pathway early post-injury, growing more prominent by the late phase, likely due to its role in neuroinflammation and cell death after ABI (6,8). Conversely, the serotonin pathway produces serotonin and melatonin, which regulate mood and circadian rhythms (9). Reduced levels of these metabolites may contribute to cognitive deficits potentially worsening rehabilitation and outcomes (10).

**Lactic acid** emerged as a key feature, distinguishing patients with favorable outcomes from those with unfavorable outcomes in early serum samples. Lactate levels were elevated in the unfavorable outcome group. Following brain injury, restricted blood flow causes cerebral hypoxia, driving neurons to anaerobic metabolism and increasing lactate production (11). Prior studies confirm elevated lactate in both animal models and ABI patients, reflecting injury severity (12–14). High lactate levels induce metabolic acidosis, contributing to cellular damage, mitochondrial dysfunction, and excitotoxicity, which exacerbate tissue injury (15). In aSAH, elevated lactate levels have been shown to predict mortality, supporting its role as a prognostic biomarker (13) Similarly, in patients with TBI, elevated lactate concentrations are strongly associated with higher short-term mortality. Furthermore, lactate appears to be a reliable predictor of 90-day mortality in patients with mild to moderate TBI (GCS ≥9) but not in those with severe TBI (GCS <8) (16). Our findings align with this evidence: higher early lactate levels signal more severe ABI, correlating with reduced recovery potential and unfavorable outcomes at three months.

**Metabolites of late time point**

The late-phase model integrated eight metabolites—prostaglandin J2, creatinine, gamma-linolenic acid, propionate, 3-hydroxy-3-methylglutarate, N-acetyl-L-alanine, N-alpha-acetyl-L-asparagine, and uridine—achieving AUC of 94.4%. These markers collectively reflect a dynamic shift in the metabolic landscape following ABI, emphasizing inflammatory modulation, systemic physiological responses, and adaptive metabolic processes that evolve as the injury progresses beyond the acute phase.

**Prostaglandin J2** (PGJ2) levels were inversely linked to poorer outcomes in late samples, with higher levels in patients achieving favorable 3-month outcomes than unfavorable. Neuroinflammation, a key response to brain injury (17), activates glial cells, releasing prostaglandins (18). Prostaglandin D2 (PGD2), predominant in the brain, rises significantly post-injury and degrades into PGJ2 (19). In vitro, PGJ2 promotes neuronal death and oxidative stress (20,21), increasing after IS and TBI (21). In vivo, it reduces edema in IS (22) and cell death in ICH (23). Our finding of favorable outcome with elevated PGJ2 aligns with these neuroprotective effects, offering an interesting prognostic insight, as prior brain injury studies rarely emphasize prostaglandins as biomarkers.

**Creatinine** levels were lower in patients with unfavorable outcomes compared to those with favorable outcomes in late serum samples. Brain injury disrupts cerebral autoregulation, heightening sympathetic tone and glomerular filtration rate (GFR), which reduces circulating creatinine (24). This autonomic dysfunction, coupled with a cytokine-mediated renal stress response, may alter kidney function post-ABI (25). Although acute kidney injury (AKI) occurs in only ~10% of TBI patients and less among the critically ill (26), elevated GFR after stroke correlates with sarcopenia, prolonged hospitalization, reduced discharge likelihood, and complications like dysphagia (27). Our findings align with this, as lower creatinine (indicating higher GFR) marked poorer outcomes. This may reflect preexisting low muscle mass, reduced protein intake, inactivity, or increased renal clearance and fluid load from severe ABI interventions (25,28).

**Gamma-linolenic acid** (GLA), an omega-6 fatty acid, exhibits anti-inflammatory and neuroprotective properties critical to post-ABI recovery. GLA generates anti-inflammatory mediators, such as prostaglandin E1 (PGE1) and 15-(S)-hydroxy-8,11,13-eicosatrienoic acid (15-HETrE), via di-homo-gamma-linolenic acid (DGLA) metabolism, competing with arachidonic acid (AA) pathways that produce pro-inflammatory prostaglandins, thromboxanes, and leukotrienes (29). This competition reduces AA-derived inflammatory agents, while GLA also mitigates reactive oxygen species (ROS), a driver of neurodegeneration after ABI (30). In our late-phase model, GLA levels were significantly lower in patients with unfavorable outcomes compared to those with favorable outcomes at 3-month follow-up. This reduction likely weakens GLA’s neuroprotective effects, enhancing AA metabolism and exacerbating tissue damage, as evidenced by increased AA-derived products in severe brain injuries (31). This GLA/AA imbalance aligns with prior research on GLA’s protective role, amplifying inflammation and neurodegeneration in poorer outcomes. Our findings position GLA as a potential biomarker of ABI severity, highlighting its prognostic and therapeutic relevance.

**Propionate**, a short-chain fatty acid (SCFA) produced by gut microbiota fermentation of dietary fiber, supports multiple physiological roles, including enteric smooth muscle contraction, gut epithelial barrier maintenance (32), and gluconeogenesis (33). It contributes to energy metabolism by conversion into propionyl-CoA and succinyl-CoA, a tricarboxylic acid (TCA) cycle substrate (Berg et al., 2002). In our late-phase model, propionate levels were elevated in patients with unfavorable outcomes compared to favorable outcomes at 3-month follow-up. This finding complicates prior evidence linking SCFAs and the gut-brain axis to neuroinflammatory diseases like stroke (34). Propionate bolsters BBB integrity via tight junction support, potentially reducing neuroinflammation in stroke (34). Preclinical mouse studies associate higher SCFA levels, including propionate, with improved stroke outcomes(35). Contrarily, our data suggest that ABI-specific energy depletion may drive gluconeogenesis via sympathetic activation, elevating propionate as a byproduct (36). This divergence highlights the propionate’s complex role, positioning it as a potential prognostic biomarker of ABI severity in our study. Further investigation is needed to clarify its contributions to systemic and CNS energy metabolism and its paradoxical effects in ABI pathology.

**3-Hydroxy-3-Methylglutarate** (HMG) is a compound structurally related to HMG-CoA, a central molecule in cholesterol biosynthesis (37). HMG-CoA also serves as a critical intermediate in ketogenesis (38). In our study, we identified HMG as a significant predictor of unfavorable outcomes following ABI. Several mechanisms may explain elevated HMG levels in these cases. One possible explanation is that severe ABI increases energy demand, shifting metabolism toward alternative energy pathways such as ketogenesis (39). As an intermediate in ketone body production, HMG may accumulate during this metabolic adaptation. Additionally, ABI-induced oxidative stress may impair mitochondrial function, including the enzymatic activity of HMG-CoA lyase, which catalyzes the conversion of HMG-CoA into acetoacetate. This enzymatic dysfunction could lead to an accumulation of HMG (37). Another potential factor is the increased demand for cholesterol synthesis following ABI to support the repair of cellular membranes and the restoration of damaged cells. This heightened need for cholesterol may lead to the accumulation of its metabolic precursors, including HMG (40). These findings suggest that elevated HMG levels may reflect metabolic stress, disrupt enzymatic processes, and heighten biosynthetic demands associated with severe ABI. Interestingly, the neuroprotective effects of statins, which act as HMG-CoA reductase inhibitors (41,42), introduce complexity to our findings. Statins are known to inhibit the metabolism of HMG-CoA into mevalonate (42), potentially leading to an accumulation of upstream intermediates, including HMG-CoA and possibly HMG, in the metabolic cascade. However, it remains unclear whether the mechanisms driving elevated HMG levels in our study mirror those of statin-mediated inhibition. The pathological elevation of HMG post-ABI may reflect a distinct metabolic dysregulation, unrelated to the therapeutic effects of statins. We consider it likely that HMG serves as a marker predicting poor outcomes through its involvement in complex metabolic pathways, rather than as an independent causative factor. This finding underscores the systemic nature of ABI, affecting the entire organism.

**N-Acetyl-L-Alanine** (NALA) and **N-alpha-acetyl-L-asparagine** (NALS) are acetylated derivatives of amino acids alanine and asparagine. These acetylated metabolites originate from the precursor free amino acids, alanine and asparagine. Under physiological conditions, amino acid concentrations in the brain remain largely stable (43); however, they undergo temporal variations in pathological states (44). Alanine has been identified as a diagnostic and prognostic biomarker in TBI and IS (45,46). Additionally, previous studies have highlighted asparagine as a significant metabolite for distinguishing between IS and ICH (47). Both NALA and NALS have significantly higher levels in patients with unfavorable outcomes in blood samples of the late time point. There are no previous studies highlighting specifically these two metabolites in the field of ABI. However, it is reasonable to assume that as the concentrations of free amino acids decline following brain injury, the metabolic balance shifts toward their acetylated derivatives. The role of NALA and NALS in the context of ABIs is not well understood. Although, there is evidence supporting the role of other acetylated AAs in the field, such as N-Acetyl-L-Leucine (NALL) improves functional recovery after TBI (48) as well as N-Acetylcysteine (NAC), which is recognized as a neuroprotective agent in preclinical studies on ABI due to its ability to reduce neuroinflammation (49). Possibly, NALA and NALS are present at higher concentrations in patients within the unfavorable cohort, as the body may attempt a neuroprotective response in cases of more severe ABI and, consequently, more intense inflammation. These markers represent intriguing targets for future research, both as potential prognostic indicators and as therapeutic targets.

**L-Methionine**: In our cohort, a pathway of two essential amino acids, cysteine and methionine is enriched in patients with ABI. It’s one of the most enriched pathways when we examined the later time point metabolites. Methionine levels seem to be higher in patients suffering from unfavorable outcomes. As we know, methionine is a precursor of homocysteine after methylation pathway. Additionally, it is a marker of the global methylation status of the body (50). Existing literature provides evidence that higher levels of homocysteine have a significant contribution to the risk of IS and aSAH (51,52). In addition, elevated levels of homocysteine seem to predict mortality and worse outcomes of stroke patients (53). In the rodent study, oxidation of methionine seems to be associated with higher inflammation and exacerbation of brain injury after the reperfusion treatment (54). We can create a hypothesis that when a patient has higher levels of methionine in the blood there is a higher likelihood of both methionine being oxidized thus leading to greater inflammation and at the same time producing more homocysteine which is associated with both neurovascular inflammation and increased ROS production leading worse outcome. It is known that excessive dietary intake of methionine can increase homocysteine levels (55). The enriched metabolic pathway identified in our study supports this theory in this context, and our findings are therefore consistent with previous studies. This positions methionine as a potential biomarker for predicting outcomes in ABI.

**References**

1. Cansev M, Wurtman RJ, Sakamoto T, Ulus IH. Oral administration of circulating precursors for membrane phosphatides can promote the synthesis of new brain synapses. Alzheimers Dement. 2008 Jan;4(1 Suppl 1):S153-168.

2. Cansev M, Watkins CJ, van der Beek EM, Wurtman RJ. Oral uridine-5’-monophosphate (UMP) increases brain CDP-choline levels in gerbils. Brain Res. 2005 Oct 5;1058(1–2):101–8.

3. Başkaya MK, Doğan A, Rao AM, Dempsey RJ. Neuroprotective effects of citicoline on brain edema and blood-brain barrier breakdown after traumatic brain injury. J Neurosurg. 2000 Mar;92(3):448–52.

4. Dempsey RJ, Raghavendra Rao VL. Cytidinediphosphocholine treatment to decrease traumatic brain injury-induced hippocampal neuronal death, cortical contusion volume, and neurological dysfunction in rats. J Neurosurg. 2003 Apr;98(4):867–73.

5. Lovelace MD, Varney B, Sundaram G, Lennon MJ, Lim CK, Jacobs K, et al. Recent evidence for an expanded role of the kynurenine pathway of tryptophan metabolism in neurological diseases. Neuropharmacology. 2017 Jan 1;112:373–88.

6. Yan EB, Frugier T, Lim CK, Heng B, Sundaram G, Tan M, et al. Activation of the kynurenine pathway and increased production of the excitotoxin quinolinic acid following traumatic brain injury in humans. J Neuroinflammation. 2015 May 30;12:110.

7. Ormstad H, Verkerk R, Aass HCD, Amthor KF, Sandvik L. Inflammation-induced catabolism of tryptophan and tyrosine in acute ischemic stroke. J Mol Neurosci. 2013 Nov;51(3):893–902.

8. Brouns R, Verkerk R, Aerts T, De Surgeloose D, Wauters A, Scharpé S, et al. The role of tryptophan catabolism along the kynurenine pathway in acute ischemic stroke. Neurochem Res. 2010 Sep;35(9):1315–22.

9. Richard DM, Dawes MA, Mathias CW, Acheson A, Hill-Kapturczak N, Dougherty DM. L-Tryptophan: Basic Metabolic Functions, Behavioral Research and Therapeutic Indications. Int J Tryptophan Res. 2009 Mar 23;2:45–60.

10. Zahar S, Schneider N, Makwana A, Chapman S, Corthesy J, Amico M, et al. Dietary tryptophan-rich protein hydrolysate can acutely impact physiological and psychological measures of mood and stress in healthy adults. Nutr Neurosci. 2023 Apr;26(4):303–12.

11. Baranovicova E, Kalenska D, Kaplan P, Kovalska M, Tatarkova Z, Lehotsky J. Blood and Brain Metabolites after Cerebral Ischemia. Int J Mol Sci. 2023 Dec 9;24(24):17302.

12. Yan EB, Hellewell SC, Bellander BM, Agyapomaa DA, Morganti-Kossmann MC. Post-traumatic hypoxia exacerbates neurological deficit, neuroinflammation and cerebral metabolism in rats with diffuse traumatic brain injury. J Neuroinflammation. 2011 Oct 28;8(1):147.

13. Dijkland S, Donkelaar KV, Van den Bergh W, Bakker J, Dippel D, Nijsten M, et al. Prognostic value of blood lactate and glucose levels after aneurysmal subarachnoid hemorrhage. Crit Care. 2015 Mar 16;19(1):P466.

14. Svedung Wettervik T, Engquist H, Howells T, Rostami E, Hillered L, Enblad P, et al. Arterial lactate in traumatic brain injury - Relation to intracranial pressure dynamics, cerebral energy metabolism and clinical outcome. J Crit Care. 2020 Dec;60:218–25.

15. Kraut JA, Madias NE. Lactic Acidosis. New England Journal of Medicine. 2014 Dec 11;371(24):2309–19.

16. Martin-Rodriguez F, Sanz-Garcia A, Lopez-Izquierdo R, Delgado Benito JF, Martínez Fernández FT, Otero de la Torre S, et al. Prehospital Lactate Levels Obtained in the Ambulance and Prediction of 2-Day In-Hospital Mortality in Patients With Traumatic Brain Injury. Neurology. 2024 Aug 27;103(4):e209692.

17. Werner C, Engelhard K. Pathophysiology of traumatic brain injury. British Journal of Anaesthesia. 2007 Jul 1;99(1):4–9.

18. Bernardo A, Ajmone-Cat MA, Levi G, Minghetti L. 15-deoxy-delta12,14-prostaglandin J2 regulates the functional state and the survival of microglial cells through multiple molecular mechanisms. J Neurochem. 2003 Nov;87(3):742–51.

19. Ricciotti E, FitzGerald GA. Prostaglandins and Inflammation. Arterioscler Thromb Vasc Biol. 2011 May;31(5):986–1000.

20. Kondo M, Oya-Ito T, Kumagai T, Osawa T, Uchida K. Cyclopentenone Prostaglandins as Potential Inducers of Intracellular Oxidative Stress *. Journal of Biological Chemistry. 2001 Apr 13;276(15):12076–83.

21. Liu H, Li W, Ahmad M, Rose ME, Miller TM, Yu M, et al. Increased generation of cyclopentenone prostaglandins after brain ischemia and their role in aggregation of ubiquitinated proteins in neurons. Neurotox Res. 2013 Aug;24(2):191–204.

22. Nicholson JD, Puche AC, Guo Y, Weinreich D, Slater BJ, Bernstein SL. PGJ2 Provides Prolonged CNS Stroke Protection by Reducing White Matter Edema. PLoS One. 2012 Dec 20;7(12):e50021.

23. Zhao X, Zhang Y, Strong R, Grotta JC, Aronowski J. 15d-Prostaglandin J2 Activates Peroxisome Proliferator-Activated Receptor-γ, Promotes Expression of Catalase, and Reduces Inflammation, Behavioral Dysfunction, and Neuronal Loss after Intracerebral Hemorrhage in Rats. J Cereb Blood Flow Metab. 2006 Jun 1;26(6):811–20.

24. Robba C, Banzato E, Rebora P, Iaquaniello C, Huang CY, Wiegers EJA, et al. Acute Kidney Injury in Traumatic Brain Injury Patients: Results From the Collaborative European NeuroTrauma Effectiveness Research in Traumatic Brain Injury Study. Critical Care Medicine. 2021 Jan;49(1):112.

25. Vlieger GD, Meyfroidt G. Kidney Dysfunction After Traumatic Brain Injury: Pathophysiology and General Management. Neurocritical Care. 2022 Nov 2;38(2):504.

26. De Rosa S, Battaglini D, Robba C. Kidney dysfunction after acute brain injury. Nephrology Dialysis Transplantation. 2024 Feb 1;39(2):170–3.

27. Yoshimura Y, Wakabayashi H, Nagano F, Bise T, Shimazu S, Shiraishi A. Elevated Creatinine-Based Estimated Glomerular Filtration Rate is Associated with Increased Risk of Sarcopenia, Dysphagia, and Reduced Functional Recovery after Stroke. Journal of Stroke and Cerebrovascular Diseases. 2021 Feb 1;30(2):105491.

28. Thongprayoon C, Cheungpasitporn W, Srivali N, Ungprasert P, Kittanamongkolchai W, Kashani K. The impact of fluid balance on diagnosis, staging and prediction of mortality in critically ill patients with acute kidney injury. J Nephrol. 2016 Apr;29(2):221–7.

29. Sergeant S, Rahbar E, Chilton FH. Gamma-linolenic acid, Dihommo-gamma linolenic, Eicosanoids and Inflammatory Processes. Eur J Pharmacol. 2016 Aug 15;785:77–86.

30. Youn K, Lee S, Jun M. *Gamma*-linolenic acid ameliorates Aβ-induced neuroinflammation through NF-κB and MAPK signalling pathways. Journal of Functional Foods. 2018 Mar 1;42:30–7.

31. Valero-Hernandez E, Tremoleda JL, Michael-Titus AT. Omega-3 Fatty Acids and Traumatic Injury in the Adult and Immature Brain. Nutrients. 2024 Nov 30;16(23):4175.

32. Killingsworth J, Sawmiller D, Shytle RD. Propionate and Alzheimer’s Disease. Front Aging Neurosci [Internet]. 2021 Jan 11 [cited 2025 Mar 6];12. Available from: https://www.frontiersin.org/journals/aging-neuroscience/articles/10.3389/fnagi.2020.580001/full

33. Ringer AI. THE CHEMISTRY OF GLUCONEOGENESIS: I. THE QUANTITATIVE CONVERSION OF PROPIONIC ACID INTO GLUCOSE. Journal of Biological Chemistry. 1912 Sep 1;12(3):511–5.

34. Hoyles L, Snelling T, Umlai UK, Nicholson JK, Carding SR, Glen RC, et al. Microbiome–host systems interactions: protective effects of propionate upon the blood–brain barrier. Microbiome. 2018 Mar 21;6(1):55.

35. Lee J, d’Aigle J, Atadja L, Quaicoe V, Honarpisheh P, Ganesh BP, et al. Gut Microbiota-Derived Short-Chain Fatty Acids Promote Post-Stroke Recovery in Aged Mice. Circ Res. 2020 Jul 31;127(4):453–65.

36. Wilmore DW, Long JM, Mason AD, Skreen RW, Pruitt BA. Catecholamines: Mediator of the Hypermetabolic Response to Thermal Injury. Ann Surg. 1974 Oct;180(4):653–68.

37. Friesen JA, Rodwell VW. The 3-hydroxy-3-methylglutaryl coenzyme-A (HMG-CoA) reductases. Genome Biol. 2004;5(11):248.

38. Laffel L. Ketone bodies: a review of physiology, pathophysiology and application of monitoring to diabetes. Diabetes/Metabolism Research and Reviews. 1999;15(6):412–26.

39. Puchowicz MA, Emancipator DS, Xu K, Magness DL, Ndubuizu OI, Lust WD, et al. Adaptation to Chronic Hypoxia During Diet-Induced Ketosis. In: Oxygen Transport to Tissue XXVI [Internet]. Springer, Boston, MA; 2005 [cited 2025 Mar 7]. p. 51–7. Available from: https://link-springer-com.ezproxy.utu.fi/chapter/10.1007/0-387-26206-7_8

40. Dietschy JM, Turley SD. Thematic review series: Brain Lipids. Cholesterol metabolism in the central nervous system during early development and in the mature animal. Journal of Lipid Research. 2004 Aug 1;45(8):1375–97.

41. Fisher M, Moonis M. Neuroprotective Effects of Statins: Evidence from Preclinical and Clinical Studies. Curr Treat Options Cardio Med. 2012 Jun 1;14(3):252–9.

42. Kosowski M, Smolarczyk-Kosowska J, Hachuła M, Maligłówka M, Basiak M, Machnik G, et al. The Effects of Statins on Neurotransmission and Their Neuroprotective Role in Neurological and Psychiatric Disorders. Molecules. 2021 May 11;26(10):2838.

43. Robinson N, Williams CB. Amino acids in human brain. Clinica Chimica Acta. 1965 Sep 1;12(3):311–7.

44. Amorini AM, Lazzarino G, Di Pietro V, Signoretti S, Lazzarino G, Belli A, et al. Severity of experimental traumatic brain injury modulates changes in concentrations of cerebral free amino acids. Journal of Cellular and Molecular Medicine. 2017;21(3):530–42.

45. Tao S, Xiao X, Li X, Na F, Na G, Wang S, et al. Targeted metabolomics reveals serum changes of amino acids in mild to moderate ischemic stroke and stroke mimics. Front Neurol. 2023 Apr 14;14:1153193.

46. Thomas I, Dickens AM, Posti JP, Czeiter E, Duberg D, Sinioja T, et al. Serum metabolome associated with severity of acute traumatic brain injury. Nature Communications [Internet]. 2022 [cited 2023 Nov 24];13. Available from: https://www.ncbi.nlm.nih.gov/pmc/articles/PMC9090763/

47. Hu Z, Zhu Z, Cao Y, Wang L, Sun X, Dong J, et al. Rapid and Sensitive Differentiating Ischemic and Hemorrhagic Strokes by Dried Blood Spot Based Direct Injection Mass Spectrometry Metabolomics Analysis. J Clin Lab Anal. 2016 Jun 9;30(6):823–30.

48. Hegdekar N, Lipinski MM, Sarkar C. N-Acetyl-l-leucine improves functional recovery and attenuates cortical cell death and neuroinflammation after traumatic brain injury in mice. Sci Rep. 2021 Apr 29;11:9249.

49. Khan M, Sekhon B, Jatana M, Giri S, Gilg AG, Sekhon C, et al. Administration of N-acetylcysteine after focal cerebral ischemia protects brain and reduces inflammation in a rat model of experimental stroke. Journal of Neuroscience Research. 2004;76(4):519–27.

50. Navik U, Sheth VG, Khurana A, Jawalekar SS, Allawadhi P, Gaddam RR, et al. Methionine as a double-edged sword in health and disease: Current perspective and future challenges. Ageing Research Reviews. 2021 Dec 1;72:101500.

51. Rudreshkumar KJ, Majumdar V, Nagaraja D, Christopher R. Relevance of plasma levels of free homocysteine and methionine as risk predictors for ischemic stroke in the young. Clinical Nutrition. 2018 Oct 1;37(5):1715–21.

52. Wei S, Yuan X, Li D, Guo X, Guan S, Xu Y. Homocysteine Levels Are Associated With the Rupture of Intracranial Aneurysms. Front Neurosci. 2022 Jul 14;16:945537.

53. Shi M, Zheng J, Liu Y, Mao X, Wu X, Chu M, et al. Folate, Homocysteine, and Adverse Outcomes After Ischemic Stroke. J Am Heart Assoc. 2024 Sep 18;13(19):e036527.

54. Gu SX, Blokhin IO, Wilson KM, Dhanesha N, Doddapattar P, Grumbach IM, et al. Protein methionine oxidation augments reperfusion injury in acute ischemic stroke. JCI Insight [Internet]. 2016 May 19 [cited 2025 Mar 7];1(7). Available from: https://insight.jci.org/articles/view/86460

55. Williams KT, Schalinske KL. Homocysteine metabolism and its relation to health and disease. BioFactors. 2010;36(1):19–24.
